# *Cr3a*, a candidate gene conferring fruit cracking resistance, was fine‐mapped in an introgression line of *Solanum lycopersicum* L.

**DOI:** 10.1111/tpj.70184

**Published:** 2025-05-02

**Authors:** Yifan Chen, Wenzheng Gao, Yu Zhu, Shuliang Qiu, Zhuoyao Qiu, Chenchen Dong, Ziteng Liu, Yongchen Du, Junming Li, Zejun Huang, Xin Li, Lei Liu, Liwang Liu, Xiaoxuan Wang

**Affiliations:** ^1^ State Key Laboratory of Vegetable Biobreeding, Institute of Vegetables and Flowers Chinese Academy of Agricultural Sciences Beijing 100081 China; ^2^ College of Horticulture Nanjing Agricultural University Nanjing 210095 China

**Keywords:** tomato, fruit cracking, *Cr3a*, map‐based cloning, gene editing, transcriptome, metabolome

## Abstract

In the cultivation and production of tomato (*Solanum lycopersicum* L.), fruit cracking is a prevalent and detrimental issue that significantly impacts the esthetic quality and commercial value of the fruit. The complexity of the trait has resulted in a slow advancement in research aimed at identifying genes that influence tomato fruit cracking and the underlying regulatory mechanisms. In this study, a sub‐introgression population for tomato crack‐resistant fruit has been constructed from the cross between *S. lycopersicum 1052* and *Solanum pennellii LA0716*, followed by 11 generations of selfing. Utilizing specifically designed InDel markers, the tomato crack‐resistant gene, *Cr3a*, was fine‐mapped, cloned, and its functionality was confirmed through transgenic and gene‐knockout approaches. The precise localization of *Cr3a* was delineated to a 30 kb genomic region on chromosome 3, corresponding to the gene *Sopen03g034650* in *S. pennellii* and *Solyc03g115660.3* in the *Heinz1706* variety. An integrated transcriptomic and metabolomic analysis of fruits with and without the *Cr3a* gene was finally conducted to elucidate the intricate regulatory mechanisms associated with *Cr3a*. The findings revealed a molecular regulatory network for tomato fruit crack resistance, characterized by 7 key metabolites, 13 pivotal genes, and 4 critical pathways: the phenylpropanoid biosynthesis pathway, the phenylalanine, tyrosine, and tryptophan biosynthesis pathway, the linolenic acid metabolism pathway, and the cysteine and methionine metabolism pathway. In summary, this research provides novel insights into the molecular underpinnings of tomato fruit crack resistance and holds substantial promise for accelerating the molecular breeding of tomatoes with enhanced fruit crack resistance.

## Significance Statement

This study has fine‐mapped the tomato fruit crack resistance gene *Cr3a* to a 30 kb genomic region on chromosome 3. Through transgenic overexpression and gene knockout, *Sopen03g034650* from *S. pennellii* has been verified as the tomato fruit crack resistance gene *Cr3a*. This provides new insights into the molecular underpinnings of tomato fruit crack resistance and accelerates breeding.

## INTRODUCTION

Tomatoes (*Solanum lycopersicum* L.), ranking as the world's second most important vegetable crop following potatoes, are celebrated for their robust adaptability and ease of cultivation, which contribute to their substantial annual yield. In 2022, the global production of fresh tomatoes was estimated at approximately 186 million tons (FAO, 2024), highlighting their critical role in global food security and nutritional well‐being, given their nutritionally balanced profile, such as significant levels of vitamins A and C.

Throughout the process of cultivated tomato breeding, extensive intraspecific hybridization has been practiced, prioritizing traits for high yield and superior quality. However, this selective breeding has inadvertently led to the loss of numerous beneficial alleles that confer resistance to both biotic and abiotic stresses, progressively narrowing the genetic base. In contrast, wild tomato species have accumulated a plethora of beneficial alleles throughout their evolutionary journey, offering a rich resource for the genetic enhancement of cultivated varieties (Qiu, 2012). Despite the potential of these wild relatives, the conventional method of utilizing materials from multi‐generational hybridization between cultivated and wild species does not fully encapsulate the beneficial alleles of the wild forms. To address this limitation, introgression lines have been developed. These lines integrate the complete genomes of wild species within the genetic context of cultivated varieties, providing not only a valuable resource for the systematic and targeted mining of beneficial alleles from wild species but also a consistent genetic background for breeding programs.

Fruit cracking in tomatoes is a prevalent issue in production, leading to accelerated water loss, reduced shelf life, and a significant impact on fruit quality and commercial value. While certain agronomic practices, such as the application of organic fertilizers, precise water management, regulation of soil and air humidity, foliar application of calcium and gibberellins, minimizing diurnal temperature fluctuations, and timely harvesting, can mitigate fruit cracking to some extent, none provide a definitive solution (Dorais et al., 2004; Peet, 1992). Consequently, cloning of genes that confer resistance to fruit cracking in tomato germplasm is of paramount importance for accelerating the development of tomato varieties with enhanced resistance to cracking.

The etiology of fruit cracking and the mechanisms of cracking resistance (CR) in tomatoes have been subjects of extensive research. Although there is a general consensus that fruit cracking is associated with the properties of the cuticular membrane, such as thickness and toughness, the specific mechanisms underlying this phenomenon remain a topic of debate among researchers (Emmons & Scott, 1997; Lee et al., 2014; Matas et al., 2004; Scott et al., 2006). It is widely acknowledged that the genetic architecture of fruit CR in tomatoes is highly complex, being a quantitative trait governed by multiple quantitative trait loci (QTLs). Furthermore, different types of cracking, such as longitudinal cracking, transversal cracking, ring cracking, and irregular cracking are influenced by distinct QTLs, each contributing a minor effect but being highly sensitive to environmental factors, making it difficult to select the CR traits directly in breeding (Wang et al., 2021). Studies on the genetic regulation of fruit cracking commenced in the 1950s, yet progress has been slow, with inconsistencies in the genetic research findings due to the use of different plant materials. Early genetic studies, such as those by Reynard (1951) and Hudson (1956), postulated that resistance to longitudinal cracking in tomatoes is controlled by two recessive genes. Building upon this research foundation, Young (1959) conducted an in‐depth study on the genetic effects of tomato resistance to transversal cracking and designated the aforementioned two recessive genes as *rl* and *cr*. Avdeev (1980), after investigating the trait of tomato resistance to ring cracking, discovered that this trait is governed by a partially dominant gene *Rc*. Abbott et al. (1986) further reported variations in fruit CR among different tomato varieties, noting that peach tomatoes and cherry tomatoes possess stronger resistance to cracking than common tomatoes. With the advancement of molecular biology techniques and utilization of various tomato germplasm resources, an increasing number of genes and QTLs associated with tomato CR have been accurately identified and characterized. For instance, Moctezuma et al. (2003) first reported a correlation between tomato fruit CR and the β‐galactosidase gene *TBG6*, where suppression of *TBG6* gene expression significantly increased the rate of tomato fruit cracking. Capel et al. (2017) utilized a set of recombinant inbred line populations expressing the CR trait in wild currant tomato (*Solanum pimpinellifolium*) and identified QTLs associated with tomato fruit cracking on chromosomes 1, 3, 8, 10, and 12 using genetic linkage maps.

Our research group previously constructed four populations related to the tomato fruit CR trait and utilized over 1200 molecular markers to conduct genetic localization studies on the QTLs associated with tomato fruit CR using those populations. More than 10 QTLs related to tomato fruit cracking have been detected, which are located on chromosomes 3, 4, 5, 6, 7, 8, and 11, respectively. Consequently, further in‐depth research is necessary to refine the preliminary QTLs that have been localized.

In this study, a sub‐introgression population for tomato fruit CR has been constructed from the cross between *S. lycopersicum 1052* and *Solanum pennellii LA0716*, followed by 11 generations of selfing. The tomato crack‐resistant gene, *Cr3a*, was then fine‐mapped, cloned, and its functionality was confirmed through transgenic and gene‐knockout approaches. Finally, an integrated transcriptomic and metabolomic analysis of fruits with and without the *Cr3a* gene was conducted to elucidate the intricate regulatory mechanisms associated with *Cr3a*. These results provided novel insights into the molecular mechanisms of tomato fruit CR, which could be used to facilitate tomato breeding.

## RESULTS

### Fine mapping of the tomato cracking‐resistant gene Cr3a

**Figure 1:**
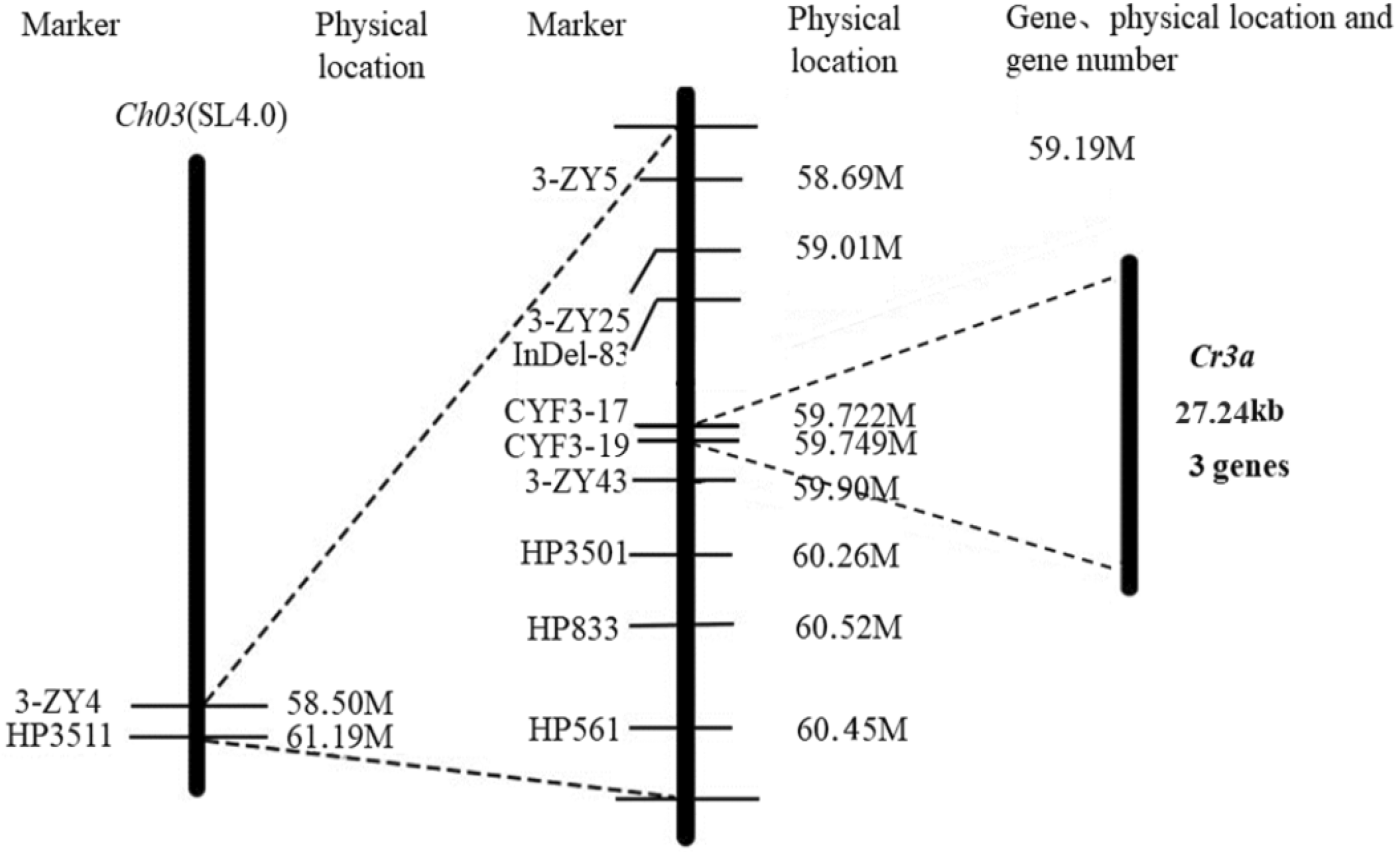
Physical map of fruit cracking‐resistant gene *Cr3a* in tomato identified by fine‐mapping using introgression lines.

The tomato cracking‐resistant gene *Cr3a* was ultimately located within an approximate 27.24 kb region between the molecular markers CYF3‐17 and CYF3‐19 at the end of chromosome 3 according to the statistical analysis on the progeny phenotypes of the plants in the constructed sub‐introgression population (Table 1; Figure 1). Compared with the genome sequence information of the Heinz 1706 and the *S. pennellii*, there are 2 (*Solyc03g115660*, *Solyc03g115680*) and 2 (*Sopen03g034650*, *Sopen03g034660*) annotated genes within the region where the gene *Cr3a* is located, respectively. Further BLAST analysis showed that the candidate gene *Solyc03g115660.3* from the Heinz 1706 genome sequence is highly homologous to the candidate gene *Sopen03g034650* from the *S. pennellii* genome sequence, and the candidate genes *Solyc03g115680.4* from the Heinz 1706 genome sequence are highly homologous to the candidate gene *Sopen03g034660* from the *S. pennellii* genome sequence (Figures S1–S4). Therefore, we used *Cr3a (650)* and *Cr3a (660)* to represent these two candidate genes for the tomato cracking resistance gene *Cr3a*, respectively. Referring to the existing tomato gene databases (NCBI, ITAG, Softberry) and annotations, *Cr3a (650)* encodes a protein kinase that is impaired in sterility induced by the natural plant resistance inducer β‐aminobutyric acid (BABA). *Cr3a (660)*, on the other hand, encodes a formin‐like protein (Table 2).

**Table 1:** Identification and statistical analysis of the phenotypic traits controlled by the fruit cracking‐resistant gene *Cr3a* in the sub‐introgression population of generations F_9_ to F_11_

F_11_	F_10_	F_9_	Polymorphic molecular markers													Phenotype	Cracking fruits number	Mature fruits number	Total fruits number	Cracking fruits rate	*P*‐value (<0.05)
			3‐ZY75	3‐ZY78	CYF3‐6	3‐ZY73	3‐ZY74	CYF3‐8	CYF3‐9	HP3495	CYF3‐15	CYF3‐17	CYF3‐18	CYF3‐19	HP557						
775	696‐156	868‐61	A	A	A	A	A	A	B	B	B	B	B	B	A	−	3	89	132	0.023	<0.0001
			A	A	A	A	A	A	A	A	A	A	A	A	A	+	25	98	123	0.203	
121	698‐199	868‐61	A	A	A	A	A	A	B	B	B	B	B	B	B	−	20	71	126	0.159	0.0166
			A	A	A	A	A	A	A	A	A	A	A	A	A	+	44	95	127	0.347	
776	702‐19	876‐4	A	A	A	A	A	A	A	A	B	B	B	B	B	−	5	89	141	0.036	0.0001
			A	A	A	A	A	A	A	A	A	A	A	A	A	+	24	95	131	0.183	
122	704‐124	876‐4	A	A	A	A	A	A	A	A	B	B	B	A	A	−	8	67	81	0.099	0.0024
			A	A	A	A	A	A	A	A	A	A	A	A	A	+	16	44	54	0.296	

A represents an exchange single plant genotype that has the same electrophoresis band as the fresh tomato KR2R144 in the parent (with phenotype of cracking‐susceptible plant, represented by +), and B represents an exchange single plant genotype that has the same electrophoresis band as the wild‐type tomato *Solanum pennellii* LA0716 in the parent (with phenotype of cracking‐resistant plant, represented by −). The gray shade indicates that the genotype in the mapping interval is heterozygous.

**Table 2:** The candidate genes *Cr3a* controlling tomato fruit cracking resistance that were identified through molecular markers assistant fine mapping in this study

Information	Candidate gene 1: *Cr3a (650)*	Candidate gene 2: *Cr3a (660)*
Gene information corresponding to the Heinz 1706 genome	*Solyc03g115660.3*	*Solyc03g115665.1* *Solyc03g115680.4*
Gene information corresponding to the *S. pennellii* genome	*Sopen03g034650*	*Sopen03g034660*
Gene sequence length predicted by the ITAG database (kb)	6.262	12.626
Gene sequence length predicted by the Softberry database (kb)	2.253	5.565
Reference sequence in the NCBI database	*XM_004235927.4*	*XM_004236434.3*
Annotation in the NCBI database	*Solanum lycopersicum* protein encoding IMPAIRED IN BABA‐INDUCED STERILITY 1 protein (LOC101257252), transcript variant X1, mRNA	*S. lycopersicum* formin‐like protein 20 (LOC101262608), transcript variant X4, mRNA
Annotation in the ITAG 2.4 database	Cell division protein kinase 12	Formin 2A
Annotation in the ITAG 4.0 database	Protein kinase superfamily protein	Formin‐like protein
Reference sequence in the Softberry database	*XP_004235975.1*	*XP_004236482.1*
Annotation in the Softberry database	Encoding IMPAIRED IN BABA‐INDUCED STERILITY 1 protein isoform X1 [*S. lycopersicum*]	Formin‐like protein 20 isoform X2 [*S. lycopersicum*]

**Figure 2:**
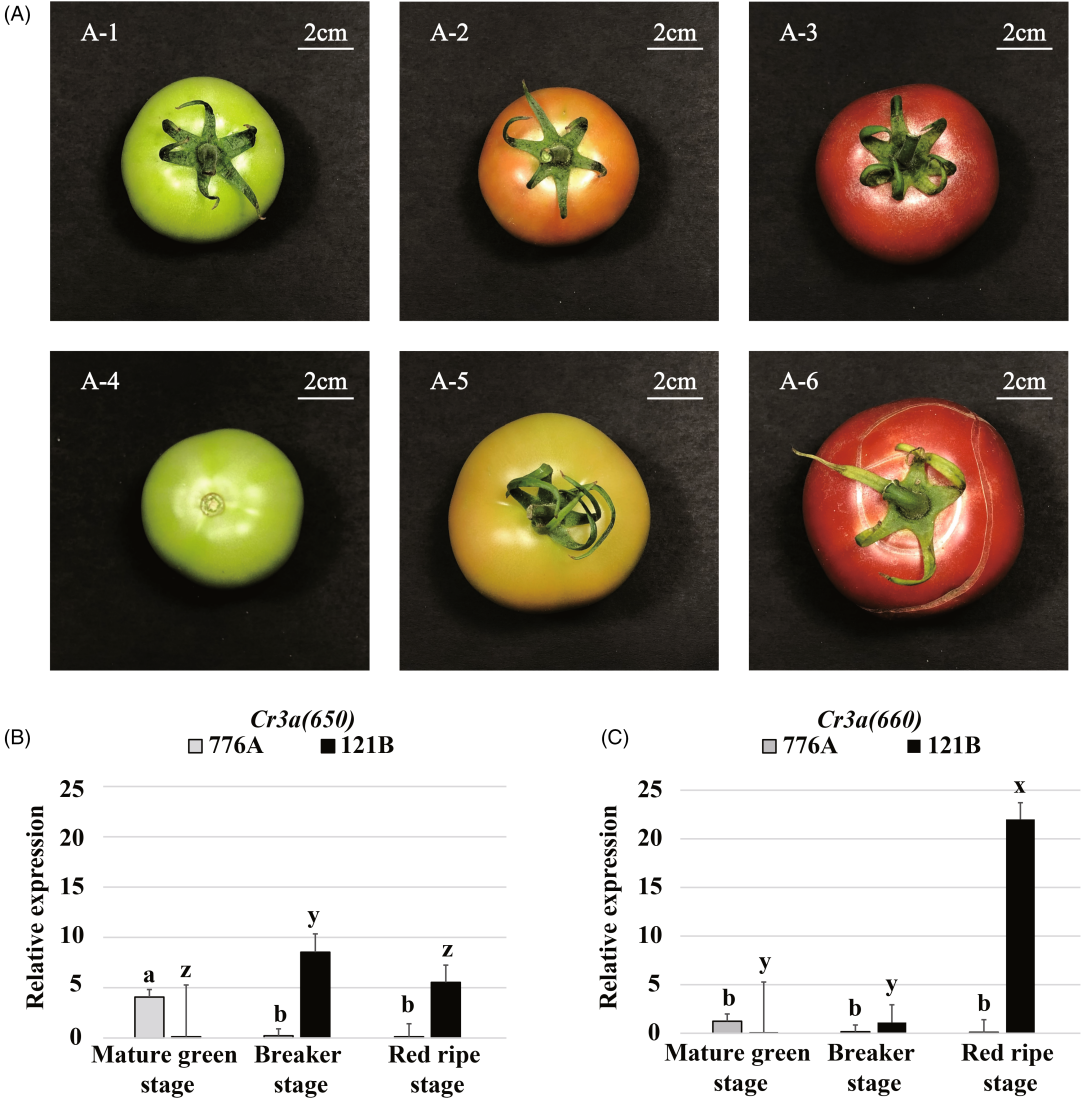
Phenotypic observation and relative expression of the two candidate genes of *Cr3a* at different developmental stages in the sub‐introgression lines constructed in this study. (A) Phenotypic observation of cracking‐resistant tomato fruits at the mature green stage (A‐1), breaker stage (A‐2), and red ripe stage (A‐3), and cracking‐susceptible tomato fruits at the mature green stage (A‐4), breaker stage (A‐5), and red ripe stage (A‐6). (B, C) Relative expression of the two candidate genes *Cr3a (650)* (B) and *Cr3a (660)* (C) controlling tomato fruit cracking resistance in the epidermis of fruit at different developmental stages. The different label (a, b, and c) on in the bar chart represent significant differences in the expression levels of corresponding genes in 776A (*P* < 0.05), with the expression levels being a > b > c; The different label (x, y, and z) on in the bar chart represent significant differences in the expression levels of corresponding genes in 121B (*P* < 0.05), with the expression levels being x > y > z.

The real‐time quantitative PCR results showed that there was a significant difference in the relative expression of *Cr3a (650)* in the pericarp of the cracking‐resistant plants 121B at different developmental stages (*P* = 0.0003), with the lower expression at the mature green stages and higher expression at the breaker stage and red ripe stage (Figure 2A,B). There was also a significant difference in the relative expression of *Cr3a (650)* in the pericarp of the cracking‐susceptible plants 776A (*P* = 0.0038) at different developmental stages, but it was only highly expressed at the mature green stages (Figure 2A,B). *Cr3a (660)* also showed significant differences in the relative expression in the pericarp of 121B and 776A at different developmental stages (*P* < 0.0001). The expression was lower at the mature green stage, higher at the crushing stage, and highest at the red fruit stage in 121B. In contrast, 776A exhibited high expression at the mature green stage (Figure 2A,C). Based on the above results, *Cr3a (650*) and *Cr3a (660)* are candidate genes for fruit cracking resistance. *Sopen03g034650* (cracking‐resistant) and *Solyc03g115660* (cracking‐susceptible) are two genotypes of Cr3a (650), while *Sopen03g034660* (cracking‐resistant) and *Solyc03g115680* (cracking‐susceptible) are two genotypes of *Cr3a (660)*.

### Transgenic and knockout of the candidate Cr3a gene

**Figure 3:**
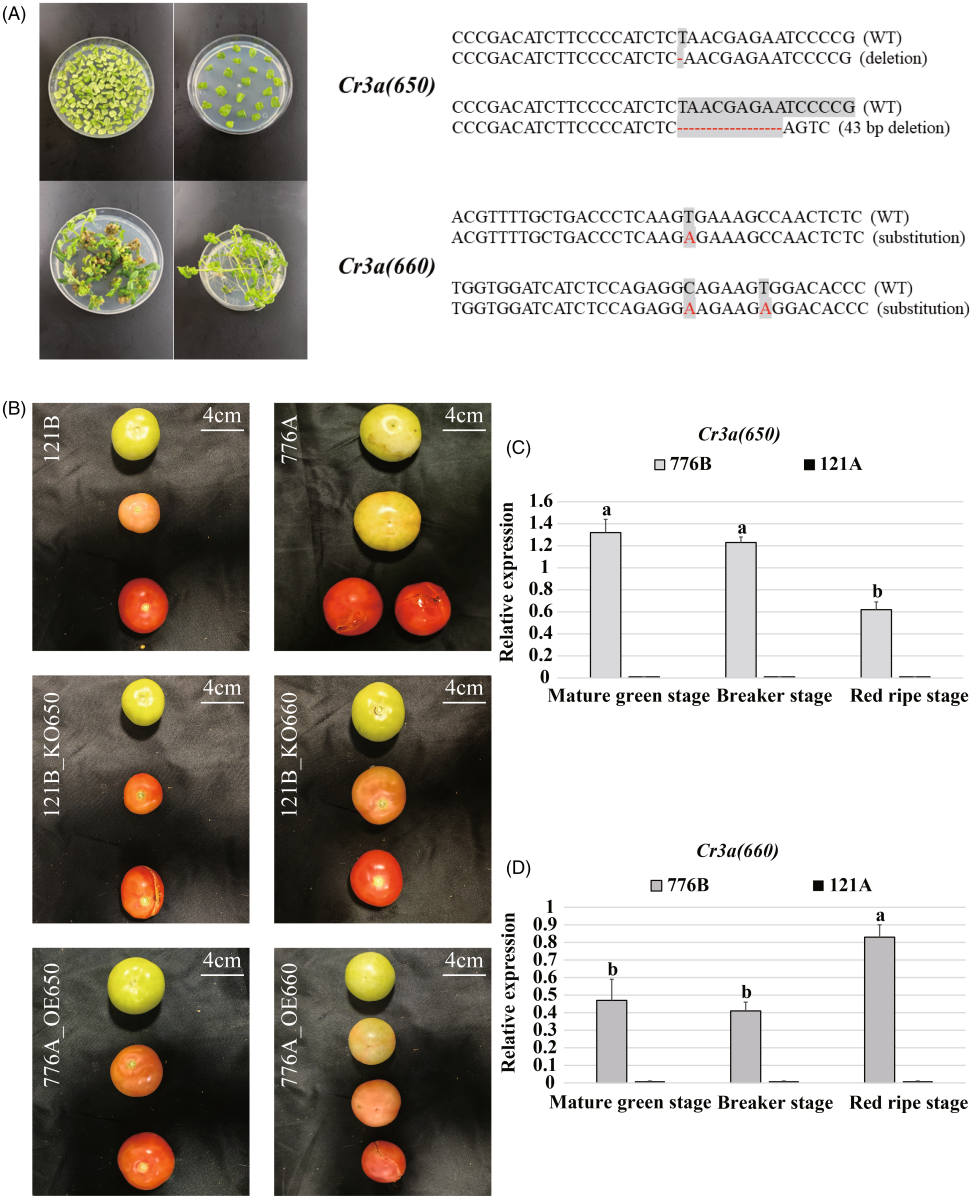
Phenotypic observation and relative expression of the two candidate genes of *Cr3a* at different developmental stages in the transgenic plant and the gene‐knockout plant constructed in this study. (A) Verification of the mutant plasmids used for *Cr3a (650)* and *Cr3a (660)* knockout by sequencing and the genetic transformation process of the transgenic tomato plant comprises the steps of co‐culturing, screening, differentiating, and rooting. (B) Phenotypes of the transgenic and gene‐knockout tomato fruits (121B: original cracking‐resistant plant 121B, 776A: original cracking‐susceptible plant 776A, 121B_KO650: cracking‐resistant plant 121B with knocked out *Cr3a (650)*, 121B_KO660: cracking‐resistant plant 121B with knocked out *Cr3a (660)*, 776A_OE650: cracking‐susceptible plant 776A transformed with *Cr3a (650)*, 776A_OE660: cracking‐susceptible plant 776A transformed with *Cr3a (660)*). (C, D) Relative expression of the two candidate genes *Cr3a (650)* (C) and *Cr3a (660)* (D) controlling tomato fruit cracking resistance in the epidermis of fruit of transgenic plant 776B and gene‐knockout plant 121A at different stages. The different label (a, b, and c) on in the bar chart represent significant differences in the expression levels of corresponding genes in 776B (*P* < 0.05), with the expression levels being a > b > c.

Genetically transformed tomato plants were obtained, and a total of 27 positive plants with successful transformation of the *Cr3a (650)* gene and 17 positive plants with successful transformation of the *Cr3a (660)* gene were detected using PCR (Figure 3A). Moreover, 23 plants without off‐target phenomena were selected based on whole‐genome sequencing, including eight plants with successful knockout of the *Cr3a (650)* gene and 15 plants with successful knockout of the *Cr3a (660)* gene. The observations of the progeny phenotypes showed that the fruits of the cracking‐resistant plant 121B exhibited cracking traits at the red ripe stage after the knockout of *Cr3a (650)*, but no cracking traits were observed after the knockout of *Cr3a (660)* (Figure 3B). Furthermore, when the two candidate genes *Cr3a (650)* and *Cr3a (660)* were separately introduced into the cracking‐susceptible plant 776A, plants with *Cr3a (650)* lost the cracking trait at the red ripe stage, while plants with *Cr3a (660)* still exhibited cracking traits at the red ripe stage (Figure 3B).

The real‐time quantitative PCR results showed the two candidate genes were almost not expressed in the gene‐knockout plant 121A constructed based on the cracking‐resistant plant 121B, while they had a certain expression in the transgenic plant 776B constructed based on the cracking‐susceptible plant 776A, further verifying the successful construction of the transgenic and the gene‐knockout plants (Figure 3C,D). Therefore, the tomato cracking‐resistant gene can ultimately be identified as *Cr3a (650)*, with the relative expression significantly lower in the pericarp of 776B at the red ripe stage compared to that in the mature green and breaker stages (*P* < 0.001), showing similarity to the relative expression pattern of the *Cr3a* gene in the cracking‐resistant plant 121B (Figure 3C).

Therefore, the tomato cracking‐resistant gene can ultimately be identified as *Cr3a* (650), with the relative expression significantly lower in the pericarp of 776B at the red ripe stage compared to that in the mature green and breaker stages (*P* < 0.001), showing similarity to the relative expression pattern of the *Cr3a* gene in the cracking‐resistant plant 121B (Figure 3C).

### Bioinformatics analysis of two genotypes of Cr3a, Sopen03g034650 and Solyc03g115660

**Figure 4:**
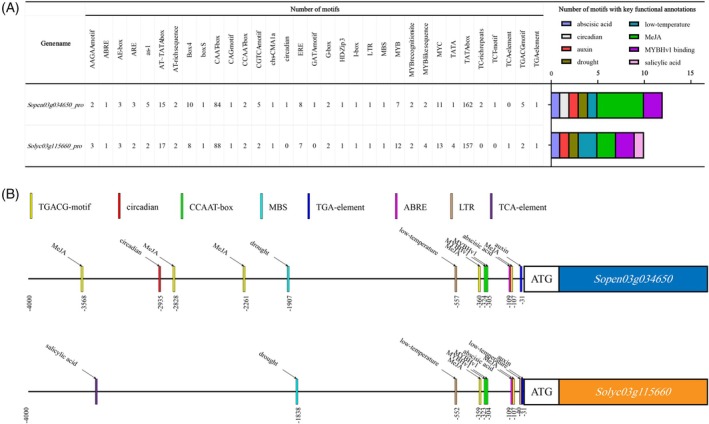
Bioinformatics analysis of *Cr3a* gene promoter. (A) The number of motifs in the *Sopen03g034650* and *Solyc03g115660* promoters (*Sopen03g034650_pro* and *Solyc03g115660_pro*), as well as the number of motifs with key functional annotations. (B) Schematic diagram of the *Sopen03g034650* and *Solyc03g115660* promoters.

Extract 4000 bp upstream of the start codon ATG in *Sopen03g034650* and *Solyc03g115660* for analysis of promoter sequences and searching for plant cis‐acting regulatory elements (Figure 4A). In the promoter analysis of the *Sopen03g034650* gene, we observed that the *Sopen03g034650* promoter contains 5 TGACG motifs (TGACG motif), annotated as Methyl Jasmonate (MeJA)‐responsiveness; 1 circular (CAAAGATTC); 2 CCAAT boxes (CAACGG), annotated as MYBHv1 binding; 1 MBS (CAACTG), annotated as drought‐inducibility; 1 TGA element (AACGAC), annotated as auxin‐responsive; 1 ABRE (ACGTG), annotated as abscisic acid responsiveness; 1 LTR (CCGAAA), annotated as low‐temperature responsiveness (Figure 4A,B). In the promoter analysis of the *Solyc03g115660* gene, we observed that the *Solyc03g115660* promoter contains two TGACG motifs (TGACG motif), annotated as Methyl Jasmonate (MeJA)‐responsiveness; two CCAAT boxes (CAACGG), annotated as MYBHv1 binding; one MBS (CAACTG), annotated as drought‐inducibility; one TGA element (AACGAC), annotated as auxin‐responsive; 1 ABRE (ACGTG), annotated as abscisic acid responsiveness; two LTR (CCGAAA), annotated as low‐temperature responsiveness; two TCA‐element (CCATCTTTTT), annotated as salicylic acid responsiveness (Figure 4A,B).

**Figure 5:**
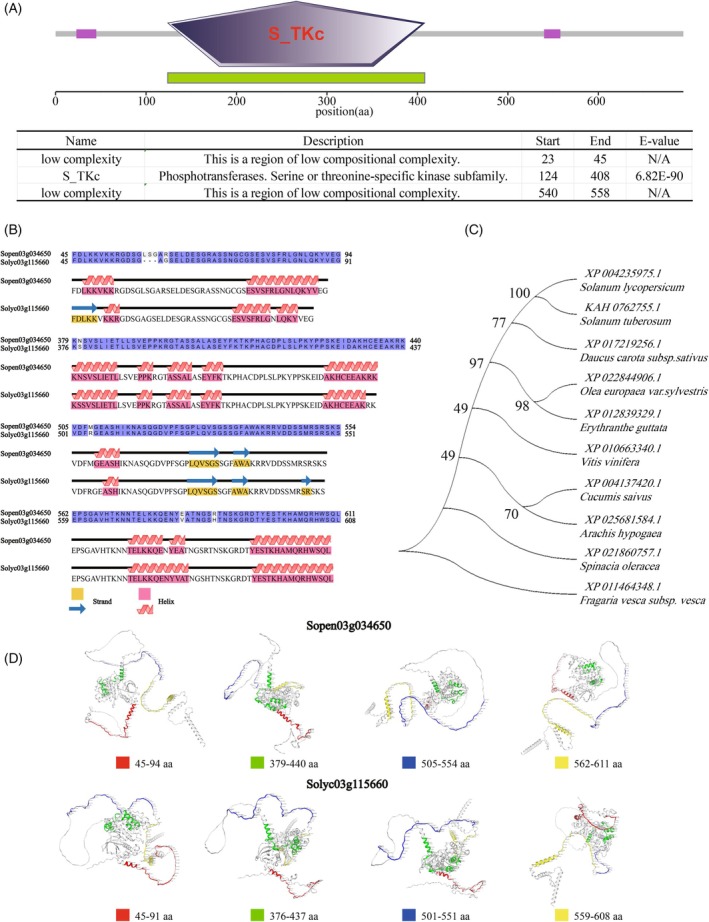
Bioinformatics analysis of the protein encoded by the *Cr3a* gene. (A) Analysis of the predicted conserved domains on the protein coded by the *Cr3a* gene. (B) Prediction of protein secondary structure changes caused by amino acid sequence variations between Sopen03g034650 and Solyc03g115660. (C) Phylogenetic tree of the protein coded by the *Cr3a* gene in tomato and its homologs in 9 plant species. (D) Prediction of the protein 3D Structure of Sopen03g034650 and Solyc03g115660, with sectional views from different directions highlighting the region of amino acid variation between Sopen03g034650 and Solyc03g115660.

Bioinformatics analysis showed that the protein encoded by the *Cr3a* gene contains 1 Serine/Threonine protein kinase, catalytic domain (S_TKc domain), lacks transmembrane domains, and is a non‐secretory protein and non‐signal peptide (Figure 5A; Figures S5 and S6).

By protein sequence alignment, we found amino acid sequence variations between the two genotypes of *Cr3a*, namely, Sopen03g034650 and Solyc03g115660. The alignment results of Solyc03g115660 with Sopen03g034650 showed a deletion between amino acid residues 58 and 60, the substitution of Arginine (R) at position 62 by Glycine (G), the substitution of Asparagine (N) at position 380 by Serine (S), the substitution of Methionine (M) at position 508 by Arginine (R), the substitution of Glutamic Acid (E) at position 582 by Valine (V), and the substitution of Arginine (R) at position 588 by Histidine (H) (as shown in Figure 5B and Figure S2). These deletions or substitutions at amino acid residues may be responsible for the differences in protein function between Sopen03g034650 and Solyc03g115660.

We conducted secondary structure analysis on Sopen03g034650 and Solyc03g115660 and found that deletions or substitutions of amino acid residues can lead to changes in the secondary structure (Figures S7 and S8). The deletion of amino acid residues from positions 58 to 60 and the substitution of Arginine (R) at position 62 with Glycine (G) may result in the conversion of an Alpha helix (LKKVKK) to an Extended strand (FDLKK) and another Alpha helix (KKR), as well as the conversion of an Alpha helix (ESVSFRLGNLQKYV) into two separate Alpha helices (ESVSFRLG and LQKY) (Figure 5B). The substitution of Asparagine (N) at position 380 with Serine (S) may cause the conversion of an Alpha helix (AKHCEEAKRK) to a shorter Alpha helix (AKHCEEAK) (Figure 5B). The substitution of Methionine (M) at position 508 with Arginine (R) may lead to the transformation of an Alpha helix (GEASH) into a shorter Alpha helix (ASH) and the emergence of an Extended strand (SR) (Figure 5B). The simultaneous substitution of Glutamic Acid (E) at position 582 with Valine (V) and Arginine (R) at position 588 with Histidine (H) may result in the conversion of two Alpha helices (TELKKQE and YEA) into a single, altered Alpha helix (TELKKQENYVAT) (Figure 5B).

We used protein structure homology modeling to predict the 3D protein structures of Sopen03g034650 and Solyc03g115660. The template A0A6J1AFV5.1.A was found to match Sopen03g034650's amino acid sequence, with a GMQE (Global Model Quality Estimate) value of 0.63 and a Sequence Identity of 75.11% (Figure S9A). The template I1JZM1.1 was found to match Solyc03g115660's amino acid sequence, with a GMQE (Global Model Quality Estimate) value of 0.65 and a Sequence Identity of 71.43% (Figure S9B).

To highlight the protein 3D structures of Sopen03g034650 and Solyc03g115660 in the regions of amino acid residue deletion and substitution, we highlighted the amino acid residues from positions 45 to 95 of Sopen03g034650 and from positions 45 to 91 of Solyc03g115660 in red. Highlight the amino acid residues from positions 379 to 440 of Sopen03g034650 and from positions 376 to 437 of Solyc03g115660 in green. Highlight the amino acid residues from positions 505 to 554 of Sopen03g034650 and from positions 501 to 551 of Solyc03g115660 in blue. Highlight the amino acid residues from positions 562 to 611 of Sopen03g034650 and from positions 559 to 608 of Solyc03g115660 in yellow (Figure 5D).

The phylogenetic tree analysis illustrated that the Cr3a proteins in tomato and potato (*Solanum tuberosum*) have the closest phylogenetic relationship, while other plants with Cr3a protein demonstrated the following phylogenetic relationships from the closest to more distant: carrot (*Daucus carota*), olive (*Olea europaea*), Mimulus pictus (*Erythranthe guttata*), grape (*Vitis vinifera*), cucumber (*Cucumis sativus*), peanut (*Arachis hypogaea*), spinach (*Spinacia oleracea*), and strawberry (*Fragaria vesca*) (Figure 5C).

### Integrative analysis of the transcriptome and metabolome in the cracking‐resistant tomato fruit and its Cr3a gene knockout counterpart

**Figure 6:**
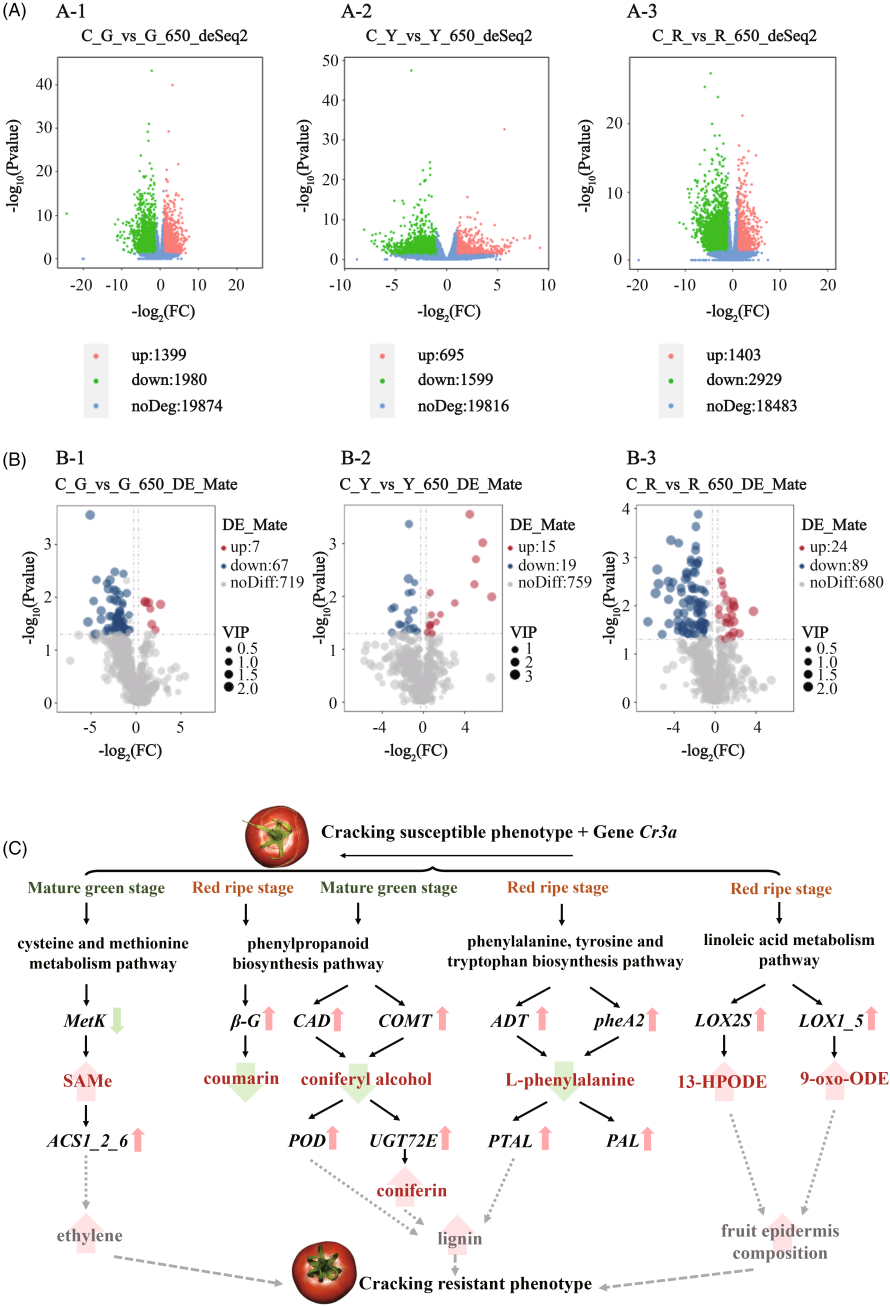
Integrative analysis of the transcriptome and metabolome in the cracking‐resistant tomato fruit and its *Cr3a* gene‐knockout counterpart. (A) Volcano plots of differentially expressed genes in samples of cracking‐resistant tomato 121B fruits and cracking‐susceptible tomato 121A fruits at the same physiological stage (A‐1: mature green stage; A‐2: breaker stage; A‐3: red ripe stage). (B) Volcano plots of differentially accumulated metabolites in samples of cracking‐resistant tomato 121B fruits and cracking‐susceptible tomato 121A fruits at the same physiological stage (B‐1: mature green stage; B‐2: breaker stage; B‐3: red ripe stage). (C) Key metabolites and the related regulatory networks affecting fruit cracking in tomatoes identified from the joint transcriptomic and metabolomic analysis at the mature green stage and the red ripe stage. 9‐oxo‐ODE, 9‐oxo‐octadecadienoic acid; 13‐HPODE, 13‐hydroperoxy‐9,11‐octadecadienoic acid; *ACS1_2_6*, 1‐aminocyclopropane‐1‐carboxylate synthase gene; *ADT*, arogenate gene; *CAD*, cinnamyl alcohol dehydrogenase gene; *COMT*, caffeic acid 3‐O‐methyltransferase gene; *LOX1_5*, linoleate 9S‐lipoxygenase gene; *LOX2S*, lipoxygenase gene; *MetK*, methionine synthase gene; *PAL*, phenylalanine ammonia‐lyase gene; *pheA2*, prephenate dehydratase gene; *POD*, peroxidase gene; *PTAL*, phenylalanine/tyrosine ammonia‐lyase gene; SAMe, S‐adenosylmethionine; *UGT72E*, coniferyl alcohol glucosyltransferase gene; *β‐G*, beta‐glucosidase gene.

DEGs and DAMs in the pericarp between the cracking‐resistant tomato plant 121B and its *Cr3a* gene‐knockout counterpart 121A (cracking‐susceptible) at different developmental stages were identified by the transcriptome and metabolome analysis. The results indicated that at the mature green stage (C_G vs. G_650), the breaker stage (C_Y vs. Y_650), and the red ripe stage (C_R vs. R_650), the number of upregulated DEGs and downregulated DEGs is 1399 and 1980, 685 and 1599, and 1403 and 2929, respectively (Figure 6A; Table S3), while the number of upregulated DAMs and downregulated DAMs is 7 and 67, 15 and 19, and 24 and 89, respectively (Figure 6B; Table S4).

Integrating the results from the transcriptome and metabolome analysis, the common enriched KEGG pathways and the DEGs and DAMs in those pathways are illustrated in Table 3. At the mature green stage (C_G vs. G_650), the pathways commonly enriched with DEGs and DAMs included the cysteine and methionine metabolism pathway (ko00270) and the phenylpropanoid biosynthesis pathway (ko00940), with 15 and 71 DEGs identified, respectively, and the corresponding DAMs being S‐adenosylmethionine (SAMe), coniferyl alcohol, and coniferin. However, at the breaker stage (C_Y vs. Y_650), no pathways were identified where DEGs and DAMs were commonly enriched. At the red ripe stage (C_R vs. R_650), the pathways commonly enriched with DEGs and DAMs included the phenylalanine, tyrosine, and tryptophan biosynthesis pathway (ko00400), the linoleic acid metabolism pathway (ko00592), and the phenylpropanoid biosynthesis pathway (ko00940), with 10, 7, and 54 DEGs identified, respectively, and the corresponding DAMs being l‐phenylalanine, quinic acid, 13‐hydroxy‐9Z,11E‐octadecadienoic acid (13‐HPODE), arachidonic acid, 9‐oxo‐octadecadienoic acid (9‐oxo‐ODE), coumarin, and syringin/eleutheroside B, with the DAM l‐phenylalanine appearing in both the phenylalanine, tyrosine, and tryptophan biosynthesis pathway and the phenylpropanoid biosynthesis pathway.

**Table 3:** The Kyoto Encyclopedia of Genes and Genomes (KEGG) pathways identified by the integrated analysis of differentially expressed genes (DEGs) and differential accumulated metabolites (DAMs) from transcriptomic and metabolomic profiling

KEGG pathway ID	KEGG pathway name	# of identified DEGs	# of identified DAMs	Information of identified DAMs and their IDs
The mature green stage (C_G vs. G_650)				
00270	Cysteine and methionine metabolism	15	1	S‐adenosylmethionine (SAMe) (C00019)
00940	Phenylpropanoid biosynthesis	71	2	Coniferyl alcohol (C00590); coniferin (C00761)
The mature green stage (C_R vs. R_650)				
00400	Phenylalanine, tyrosine and tryptophan biosynthesis	10	2	l‐phenylalanine (C00079); quinic acid (C00296)
00591	Linoleic acid metabolism	7	3	13‐hydroxy‐9Z,11E‐octadecadienoic acid (13‐HPODE) (C04717); Arachidonic acid (C00219); 9‐oxo‐octadecadienoic acid (9‐oxo‐ODE) (C14766)
00940	Phenylpropanoid biosynthesis	54	3	l‐phenylalanine (C00079); Coumarin (C05851); Syringin/eleutheroside B (C01533)

Specifically, as illustrated in Figures S10–S14 and summarized in Figure 6(C), in the pericarp of the cracking‐susceptible plant 121A at the mature green stage, the level of SAMe was significantly downregulated, influenced by the upregulation of the upstream gene methionine synthase (*MetK*, EC:2.5.1.6) and the downregulation of the downstream gene 1‐aminocyclopropane‐1‐carboxylate synthase (*ACS1_2_6*, EC:4.4.1.14). Additionally, the level of coniferyl alcohol was significantly upregulated, and the level of coniferin was significantly downregulated, influenced by the downregulation of the upstream genes cinnamyl alcohol dehydrogenase (*CAD*, EC:1.1.1.195) and caffeic acid 3‐O‐methyltransferase (*COMT*, EC:2.1.1.68), as well as the downstream genes peroxidase (*POD*, EC:1.11.1.7) and coniferyl alcohol glucosyltransferase (*UGT72E*, EC:2.4.1.111). In the pericarp of the cracking‐susceptible plant 121A at the red ripe stage, the level of l‐phenylalanine was significantly upregulated, influenced by the upregulation of the upstream genes prephenate dehydratase (*pheA2*, EC:4.2.1.51) and arogenate dehydratase (*ADT*, EC:4.2.1.91), and the downregulation of the downstream genes phenylalanine ammonia‐lyase (*PAL*, EC:4.3.1.24) and phenylalanine/tyrosine ammonia‐lyase (*PTAL*, EC:4.3.1.25). In addition, the level of coumarin was also significantly upregulated, influenced by the downregulation of the upstream gene beta‐glucosidase (*β‐G*, EC:3.2.1.21). Furthermore, the levels of 13‐HPODE and 9‐oxo‐ODE were significantly downregulated, influenced by the downregulation of the upstream genes lipoxygenase (*LOX2S*, EC:1.13.11.12) and linoleate 9S‐lipoxygenase (*LOX1_5*, EC:1.13.11.58). In addition, although the levels of quinic acid, arachidonic acid, and syringin/eleutheroside B at this stage also showed significant downregulation, they were not included in the final regulatory network for tomato cracking‐resistant due to the lack of direct changes in their upstream and downstream DEGs.

## DISCUSSION

### Fine mapping of the tomato cracking‐resistant gene Cr3a

In introgression line populations, the majority of the genetic background of the plants is uniform, with only minor variations present on a limited segment of chromosomes. This approach can effectively mitigate the interference of genetic variation and neutralize the epistatic effects of parental materials, subsequently enhancing the likelihood of identifying minor QTLs (Chen et al., 2023). Eshed and Zamir (1994) established the inaugural introgression line population in tomatoes, utilizing the wild‐type species *S. pennellii* and the processed tomato cultivar *M82* as the parental lines, which still remains the most extensively employed resource for QTL mapping and candidate gene elucidation in tomato research. In prior investigations, our research team employed the contemporary superior cultivated tomato variety *S. lycopersicum 1052* and the wild relative *S. pennellii LA0716*. Through a series of processes including hybridization, backcrossing, molecular marker‐assisted selection, selfing, and phenotypic characterization, we successfully developed sub‐introgression line populations for traits such as tomato fruit CR, diverse fruit coloration, and distinct leaf phenotypes (Chen et al., 2023; Qiu, 2012). In contrast to other introgression line populations developed for the breeding of processing or cherry tomatoes, such as *S. lycopersicoides LA2951*, *S. habrochaites LA1777*, and *S. habrochaites LYC4*, our population is tailored for fresh‐market tomatoes, encompassing a broader spectrum of accessions and featuring a greater number of smaller segments across each chromosome, which can facilitate a more precise mapping of genetic traits for fresh‐market tomatoes. Using these breeding materials, a type of tomato fruit cracking known as reticulate cracking was observed, with micro‐cracks appearing in the pericarp on the 23rd day after pollination, and during the mature green stage, cracks could form a reticulate pattern across the entire fruit surface (Cui et al., 2017). The gene controlling the reticulate cracking trait, ER4.1, was fine‐mapped at the end of chromosome 4 within a 300 kb region between the molecular marker InDel4 and InDel82, with the candidate genes speculated to be *Solyc04g082540*, *Solyc04g082630*, *Solyc04g082910*, and *Solyc04g082950* (Cui et al., 2017), which were consistent with previously reported findings (Monforte et al., 2001; Yeats et al., 2014). However, the genetic patterns of other common types of tomato fruit cracking, such as longitudinal cracking or ring cracking, have not been clearly reported, which is the issue that this study aims to address.

In the current study, a sub‐introgression population for tomato CR was constructed. Through phenotypic investigations and the InDel molecular markers‐assisted screening, the gene controlling longitudinal and ring cracking in tomato fruits, *Cr3a*, was ultimately mapped to a 30 kb region between two InDel molecular markers on chromosome 3. However, after 11 generations of population construction, the approximately 30 kb region finally localized still contained two complete gene fragments, named *Cr3a (650)* and *Cr3a (660)*, and the range could not be further narrowed down. Therefore, the functional verification using transgenic and gene‐knockout techniques was conducted to further identify the specific candidate gene.

### Functional verification of the tomato cracking‐resistant gene Cr3a

In this study, recombinant plasmids with the *Cr3a (650)* and *Cr3a (660)* gene overexpressed were constructed, and the *Agrobacterium* transformation method was used to introduce them into the cracking‐susceptible tomato plant 776A selected from the sub‐introgression population. At the same time, the CRISPR/Cas9 technology was used to knock out the *Cr3a (650)* and *Cr3a (660)* gene fragments in the cracking‐resistant tomato plant 121B, and the resulting phenotypes were identified. The results showed that the introduction of the *Cr3a (650)* gene fragment into the cracking‐susceptible tomato plant 776A alleviated the cracking trait, while the knockout of the *Cr3a (650)* gene fragment in the cracking‐resistant tomato plant 121B resulted in the appearance of the cracking trait. In the meantime, the introduction and knockout of the *Cr3a (660)* gene fragment did not produce the expected phenotypes. Additionally, the quantitative analysis of the gene fragments using RT‐PCR technology fully demonstrated that *Cr3a (650)* is the gene fragment that regulates tomato CR.

The results indicate that the expression patterns of *Cr3a* in cracking‐resistant and cracking‐susceptible materials differ during the mature green stage, breaker stage, and red ripe stage (Figure 2B,C). This suggests that it is necessary to analyze the promoter sequences and plant cis elements derived from *Sopen03g034650* and *Solyc03g115660*. The analysis results indicate that the upstream *cis*‐acting regulatory elements of Cr3a are functionally annotated with MeJA‐responsiveness, circadian control, MYBHv1 binding, drought‐inducibility, auxin‐responsive, abscisic acid responsiveness, low‐temperature responsiveness, and salicylic acid responsiveness (Figure 4A,B). The regulatory pattern of the promoter and its upstream transcription factors is still worth further research.

According to the annotations in the ITAG database, the gene fragment *Cr3a (650)* controlling the tomato CR found in this study encodes a protein kinase, cyclin‐dependent kinase 12 (CDK12), which belongs to the CDKE class, and is reported to be involved in a variety of cellular processes, including RNA processing, DNA damage response, and transcriptional regulation (Wang et al., 2023). For example, CDK12 was reported to participate in the regulation of the cell cycle by phosphorylating and activating other proteins necessary for the progression of the cell cycle, especially the transition from the G1 phase to the S phase, and can also phosphorylate components of the RNA polymerase II complex, regulate DNA transcription, and be involved in RNA processing, especially the maturation of ribosomal RNA (rRNA) and small nuclear RNA (snRNA) and other RNA molecules (Wang et al., 2023). In mammals, CDK12 is reported to be related to the cell's response to DNA damage, and its dysregulation is associated with various types of cancer, making it a potential therapeutic target for cancer treatment (Filippone et al., 2022). In plants, CDK family genes (including CDK1–CDK22) are thought to be related to the drought and salt‐alkali tolerance traits of quinoa, and this stress resistance characteristic and mechanism may also exist in other vascular plants (Wang et al., 2023). However, the other functions of CDK in plants have not been widely reported. The regulatory role of CDK on the CR trait of tomato found in this study is also the first to be reported. In addition, previous studies in Arabidopsis have reported that some gene functions were impaired in sterility induced by β‐aminobutyric acid, resulting in some mutants, among which the ibs1 mutant encoded a protein similar to CDK, and the BLAST results showed a protein similarity of over 80% (Ton et al., 2005). Nevertheless, the specific role of CDK in this process and its connection with stress resistance and CR traits are still worth further research.

### Molecular regulatory network analysis of the tomato crack resistance gene Cr3a and the crack resistance trait

To further reveal the regulatory network behind the *Cr3a* gene related to the CR trait of tomato, the cracking‐resistant tomato plant in the sub‐introgression population and the new plant constructed by knocking out the *Cr3a (650)* gene in this plant using the CRISPR/Cas9 technique were used as plant materials, and transcriptomics and metabolomics data were jointly analyzed to excavate consistently DEGs and DAMs. Through joint analysis, seven key metabolites were screened, including SAMe, coniferyl alcohol, coniferin, l‐phenylalanine, coumarin, 13‐HPODE, and 9‐oxo‐ODE, as well as 13 key regulatory upstream and downstream genes including *MetK*, *ACS1_2_6*, *CAD*, *COMT*, *POD*, *UGT72E*, *pheA2*, *ADT*, *PAL*, *PTAL*, *β‐G*, *LOX2S*, and *LOX1_5*, and the corresponding four KEGG pathways.

In the cysteine and methionine metabolism pathway, as an upstream gene regulated by SAMe, *MetK* encodes methionine synthase, which is directly involved in the synthesis of methionine. Methionine is not only a key amino acid in plant growth, development, and stress responses, but also a precursor for ethylene biosynthesis, which is a very important plant hormone that can promote fruit ripening. As a key gene in the downstream pathway of SAMe, *ACS1_2_6* is also involved in the ethylene biosynthesis pathway. It encodes an enzyme that catalyzes the conversion of SAMe into 1‐aminocyclopropane‐1‐carboxylic acid (ACC), which is subsequently converted into ethylene by 1‐aminocyclopropane‐1‐carboxylic acid oxidase (ACO) (Fatma et al., 2022). In the phenylpropanoid biosynthesis pathway and phenylalanine, tyrosine and tryptophan biosynthesis pathway, coniferyl alcohol and coniferin are precursors in the formation of plant lignin, which play an important role in the formation and structural stability of plant cell walls. However, the biochemical synthesis of lignin can lead to the deposition of russet, which in turn causes the splitting of fruits in horticultural plants (Wang et al., 2021). The upstream and downstream genes directly regulated by this process, including *CAD*, *COMT*, *POD*, and *UGT72E*, were all significantly upregulated in this study. The upstream genes directly involved in the regulation of l‐phenylalanine include *pheA2* and *ADT*, which were both significantly upregulated in the fruit of tomato varieties resistant to cracking. The *pheA2* gene encodes the prephenate dehydrogenase that catalyzes the dehydration of prephenate to produce 4‐hydroxyphenylpyruvate, an important intermediate in the synthesis of phenylalanine and tyrosine. The downstream genes include *PAL* and *PTAL*, which were also significantly upregulated in the fruit of tomato varieties resistant to cracking. They encode the phenylalanine/tyrosine ammonia‐lyase (commonly referred to as PAL or TAL), which can cleave the chemical bond between phenylalanine or tyrosine and ammonia to produce *trans*‐cinnamic acid, a key step in the synthesis of lignin and other secondary metabolites in plants (Barros & Dixon, 2020). In the linoleic acid metabolism pathway, 13‐HPODE and 9‐oxo‐ODE are products of fatty acid oxidation within plants, which are directly regulated by the upstream genes *LOX2S* and *LOX1_5*, respectively. In the fruit of tomato varieties resistant to cracking, these genes were significantly upregulated. The enzymes encoded by these genes, lipoxygenase and linoleate 9S‐lipoxygenase, belong to a class of non‐heme iron proteins. They catalyze the peroxidation of hydrogen at specific positions in polyunsaturated fatty acids, which may be related to changes in the chemical composition of the tomato pericarp. The specific mechanism remains to be further explored. In this study, the discovered key regulatory genes, such as *CAD*, *COMT*, *POD*, and *PAL*, and pathways such as phenylpropanoid biosynthesis and linoleic acid metabolism have also been reported in other studies revealing fruit cracking mechanisms based on omics methods (transcriptomics, proteomics, etc.), such as *Akebia trifoliata* (Niu et al., 2020), litchi (Wang et al., 2019), watermelon (Jiang, Tian et al., 2019; Liao et al., 2020), and pomelo (Tao et al., 2001), which are partly in line with the results obtained in this study. In addition, unlike the fruit cracking mechanisms reported in horticultural plants such as litchi (Wang et al., 2019), the regulation of the *Cr3a* gene in this study did not involve the cutin, suberin, and wax biosynthesis pathways, which echoes the previous findings of our research group that there were no significant differences in the puncture resistance and cuticle thickness between the cracking‐resistant and cracking‐susceptible tomato fruits (Zhu, 2019).

Nevertheless, in conjunction with recent research on tomato fruit cracking, the genes and related mechanisms regulating CR are not singular and may involve the variety of tomatoes as well as the type of cracking. For example, Jiang, Lopez et al. (2019) used gene editing technology to construct a transgenic tomato line (pg/exp) that simultaneously suppresses the expression of two genes, polygalacturonase (PG) and expansin (EXP), and found that its cracking rate was significantly lower than that of the wild‐type tomato (Alisa Craig, AC). In the pg/exp plant fruits, the expression of EXP and PG proteins was downregulated, the content of water‐soluble pectin in the pericarp decreased, and the content of protopectin increased, resulting in a thicker cell wall and cuticle layer, increased fruit hardness, and a reduced cracking rate of tomato fruits, while the fruit contained more soluble solids, and the quality was better (Jiang, Lopez et al., 2019). Through mRNA and lncRNA sequencing analysis, it was further reported that lncRNAs could regulate the hormone‐redox‐cell wall network, including plant hormones (auxin, ethylene) and ROS (H_2_O_2_) signal transduction, as well as many cell wall‐related mRNAs (*PG*, *EXP*, etc.) and some lncRNAs (*XLOC_16662*, *XLOC_033910*, etc.), constructing a response network for tomato fruit cracking induced by water (Xue et al., 2020), indicating the complex mechanism and regulatory network of tomato cracking. Analysis of the physiological characteristics of the tomato fruits from the cracking‐resistant and cracking‐susceptible plants in the sub‐introgression population used in the current study exhibited that the water content of the cracking‐resistant tomato fruits was significantly lower than that of the cracking‐susceptible tomato fruits, with the most significant difference at the mature green stage, but the puncture resistance and cuticle thickness of the two kinds of tomato fruits showed no significant differences in terms of fruit hardness testing and cuticle morphology observation based on paraffin sectioning (Zhu, 2019). The molecular regulatory network revealed in this study highlights the role of key metabolites and genes in tomato fruit cracking resistance, and also confirms this result. The downregulation of *LOX2S* and *LOX1_5* in cracking‐resistant fruits suggests reduced oxidative stress, which may contribute to improved cell wall stability and reduced cracking. Additionally, the upregulation of *CAD* and *PAL* in cracking‐resistant fruits indicates enhanced lignin biosynthesis, which strengthens the fruit's structural integrity. These findings provide a physiological explanation for the observed differences in fruit size and water content between cracking‐resistant and cracking‐susceptible fruits. The lower water content in cracking‐resistant fruits may reduce internal pressure, further contributing to their resistance to cracking. Although the research perspectives discussed above are different, all those studies have provided theoretical support and reserved genes and germplasm resources for the breeding of tomato varieties with excellent traits on fruit cracking resistance.

## CONCLUSION

A sub‐introgression population of fresh‐market tomatoes with the fruit cracking resistance trait was obtained in this study. Using these plant materials, the tomato cracking resistance gene *Cr3a* was fine‐mapped to a region within 30 kb at the end of chromosome 3, which includes two complete candidate genes. Through transgenic and gene‐knockout experiments, *Sopen03g034650* (*S. pennellii*)/*Solyc03g115660.3* (*Heinz1706*) was confirmed as the final corresponding gene for *Cr3a*. The gene encodes a protein kinase with one conserved domain, lacks transmembrane domains, and is a non‐secretory protein. Evolutionarily, it is most closely related to the homologous protein in potato. The expression of this gene decreases progressively during the mature green, breaker, and red ripe stages of the tomato. Joint analysis of the transcriptome and metabolome has elucidated the molecular regulatory network surrounding the *Cr3a* gene for the tomato fruit cracking resistance trait, which includes seven key metabolites (SAMe, coniferyl alcohol, coniferin, l‐phenylalanine, coumarin, 13‐HPODE, and 9‐oxo‐ODE), 13 key genes (*MetK*, *ACS1_2_6*, *CAD*, *COMT*, *POD*, *UGT72E*, *pheA2*, *ADT*, *PAL*, *PTAL*, *β‐G*, *LOX2S*, and *LOX1_5*), and four pathways (phenylpropanoid biosynthesis pathway, phenylalanine, tyrosine and tryptophan biosynthesis pathway, linoleic acid metabolism pathway, and cysteine and methionine metabolism pathway).

## MATERIALS AND METHODS

### Plant materials and construction of sub‐introgression population

The tomato materials utilized in this study included the fresh‐market tomato variety KR2R144 and the wild‐type tomato *S. pennellii* LA0716. A chromosomal segment introgression line (IL) population was constructed by backcrossing KR2R144 with *S. pennellii* LA0716 for 5 generations followed by three generations of selfing to obtain BC_5_S_3_. The line 14h616 from this population contained a segment of *Cr3a*, located at SL4.0 ch03: 58898909–61188863 (2.26 Mb), which confers resistance to fruit cracking and contributed 21.26% to the heritability (Chen et al., 2023). The resistance to fruit cracking was found to be incompletely dominant over susceptibility. Further crossbreeding of the fresh‐market tomato KR2R144 with the line 14h616 from the introgression line population yielded an F_1_ generation, leading to the development of sub‐introgression lines population through continuous selfing and the establishment of segregation populations from F_2_ to F_11_ generations from 2014 to 2020. All the above materials were planted in the greenhouses of the Institute of Vegetables and Flowers of the Chinese Academy of Agricultural Sciences in Beijing. Five plants from each sub‐introgression line population were randomly selected as a replicate for phenotypic trait investigation, and fruit cracking traits were surveyed and analyzed once the plants produced at least four trusses of fruit, and the first two trusses ripened. Based on the statistical results, exchange single plants were selected, and their seeds were collected for propagation in the following season.

### Development of polymorphic molecular markers and fine‐mapping

All seedlings were raised in 32‐hole seed trays, and at the four‐leaf stage, the apical young leaves of the tomato plants were collected. Molecular marker‐assisted selection was employed to screen for exchange single plants with the target gene and to identify the progeny for field planting. The development of polymorphic molecular markers in this study was based on the resequencing results of the genomes of the wild‐type tomato *S. pennellii* and the cultivated tomato Heinz 1706. By comparing these sequences using the genomic visualization software IGV (https://igv.org/), the insertion and deletion (InDel) sites were identified, primers were designed, and the target genes were preliminarily located using polymorphic InDel molecular markers evenly distributed across 12 chromosomes (Qiu, 2012). This process was repeated to develop and screen for polymorphic InDel markers on the target chromosome (chromosome 3) and the target segment, which were used for the fine mapping of the tomato crack resistance gene *Cr3a*. Primers were designed using the online software Primer Select (https://bio.tools/primerselect#!). The information of major InDel molecular markers developed in this study is given in Table S1. Tomato DNA was extracted using the cetyltrimethylammonium bromide (CTAB) method and checked for concentration, purity, and quality using the micro‐spectrophotometer and 3% agarose gel electrophoresis. Based on the designed InDel molecular markers, PCR amplification of tomato DNA was performed using a PTC‐200™ Peltier thermal cycler, and 8% polyacrylamide gel electrophoresis was performed to determine the genotypes of the samples or exchange single plants, with reference to the parental genotype bands. In the constructed sub‐introgression population, plants with genotype bands identical to those of the fresh tomato parent KR2R144 were denoted as “A” those with bands identical to the wild‐type tomato *S. pennellii* LA0716 were denoted as “B” and heterozygous genotype bands were indicated as “H.” The fruit cracking rate was calculated based on phenotypic investigation results. Differences in cracking rates between genotypes were tested for homogeneity using the Chi‐squared test with the PROC GENMOD procedure in SAS 9.2. A *P*‐value of less than 0.05 was considered statistically significant.

### Real time fluorescence quantitative PCR (qRT‐PCR)

The outer epidermis of the tomato fruits from cracking‐susceptible line 776A (F_11_) and cracking‐resistant line 121B (F_11_) at different developmental stages (mature green, breaker, pre‐red ripe, and red ripe) was collected, immediately frozen in liquid nitrogen, and stored at −80°C. For each genotype and developmental stage, at least 7 fruit samples from different individual plants were pooled as one biological replicate, with three biological replicates per genotype and stage. Total RNA was extracted using the Polysaccharide and Polyphenol Plant RNA Extraction Kit (Huayueyang Biotechnology Co., Beijing, China) following the manufacturer's instructions. RNA concentration, purity, and quality were assessed using a micro‐spectrophotometer and 1% agarose gel electrophoresis. Reverse transcription to cDNA was performed using the All‐In‐One 5×RT MasterMix (abm, Canada). Real‐time quantitative PCR (qPCR) was conducted using the LightCycler® 480 II instrument. The relative expression levels of the target genes were calculated using the Pfaffl method (Pfaffl, 2001), which accounts for differences in amplification efficiency and is suitable for comparing multiple developmental stages. All primer sequences used in this study are listed in Table S2A.

### Transgenic and gene knockout

Two candidate fragments (2082 and 2808 bp, respectively) of the *Cr3a* gene were amplified via PCR using gene‐specific primers *Cr3a (650)‐overexpression* F/R and *Cr3a (660)‐overexpression* F/R (Table S2B) and cloned into the pBWA(V)HS‐GLosgfp vector, respectively. The guide RNA (Table S2B) was designed to target the conserved site within the first exon of *Cr3a (650)* and *Cr3a (660)*. The sgRNA was assembled into the pHSbdcas9i vector via BsaI/Eco31I restriction enzyme sites. The pBWA(V)HS‐GLosgfp‐*Cr3a* vectors and the pHSbdcas9i vector (containing fragment encoding Cas9 protein optimized for monocot and dicot plants) were then mixed with the *Agrobacterium tumefaciens* strain GV3101 competent cells for electroporation using an electroporation cuvette, respectively. The activated *Agrobacterium* cultures were inoculated onto an LB solid medium with kanamycin and rifampicin resistance, then incubated at 30°C in the dark for 48 hours and screened for positive results using PCR and 1% agarose gel electrophoresis. Primers for colony PCR in transgenic experiments are referenced in Table S2C. Tomato explants from well‐germinated tomato seedlings with fully extended cotyledons were used for genetic transformation. The explants were infected with *Agrobacterium tumefaciens* strain GV3101 harboring the recombinant plasmids and co‐cultivated on a solid medium in the dark at 23 ± 2°C for 2 days. The callus tissue was then transferred sequentially to a selection medium and a differentiation medium and cultivated for 15–30 and 30–40 days, respectively. The differentiated seedlings were then cut off and transplanted onto a rooting medium when they reached a height of 2–3 cm and cultivated under the same conditions for 10–15 days. Transgenic positively plants were screened using PCR following the *Agrobacterium* positive detection method. Off‐target effects of gene knockout were determined according to the whole‐genome sequencing of the potential off‐target sites predicted by software.

### Bioinformatics analysis of the protein encoded by the target gene

Based on the coding sequence (CDS) and the corresponding amino acid sequence, a bioinformatics analysis of the protein encoded by the *Cr3a* gene was conducted. The physicochemical properties and primary structure of the Cr3a protein were analyzed using Expasy (http://web.expasy.org/protparam/). The Cr3a protein domains were analyzed using SMART (https://smart.embl.de). The transmembrane domains of the Cr3a protein were predicted using DeepTMHMM (https://dtu.biolib.com/DeepTMHMM). The signal peptide of the Cr3a protein was predicted using SignalIP‐5.0 (https://services.healthtech.dtu.dk/service.php?SignalP‐5.0). A phylogenetic tree was constructed for the tomato Cr3a protein and its homologous proteins from 9 closely related species using the Neighbor‐Joining method in MEGA 11.0 software (https://www.megasoftware.net/). Protein sequence queries were performed using the protein BLAST tool from NCBI (https://blast.ncbi.nlm.nih.gov/Blast.cgi?PROGRAM=blastp&PAGE_TYPE=BlastSearch&LINK_LOC=blasthome), and sequence alignment was carried out using the ClustalW method. The Bootstrap method was selected for testing the reliability of the constructed tree, with the number of tests set to 1000. The p‐distance model was used for calculating genetic distances, and the partial deletion method was chosen for handling gaps/missing data. The analysis of promoter sequences and searching for plant *cis*‐acting regulatory elements was conducted using PlantCARE (https://bioinformatics.psb.ugent.be/webtools/plantcare/html/). Protein sequence alignment of two genotypes of Cr3a, Sopen03g034650 and Solyc03g115660, was performed using the UniProt Align tool (https://www.uniprot.org/align). Protein secondary structure prediction was conducted using PSIPRED (http://bioinf.cs.ucl.ac.uk/psipred/). The prediction of the protein 3D structure was carried out using the homology‐modeling method, specifically through protein structure homology‐modeling utilizing SWISS‐MODEL (https://swissmodel.expasy.org/).

### Integrative analysis of the transcriptome and metabolome

The plant materials used in the multi‐omics analysis included the cracking‐resistant tomato fruits from the F_12_ of the sub‐introgression population with the *Cr3a* gene highly expressed at the mature green, breaker, and red ripe stages (designated as C_G, C_Y, and C_R, respectively), and their gene‐edited counterparts with the *Cr3a* gene knocked out at the mature green, breaker, and red ripe stages (designated as G_650, Y_650, and R_650, respectively). Comparisons were made within the same developmental stage as C_G versus G_650, C_Y versus Y_650, and C_R versus R_650. Each group had three replications (*n* = 3), and each replicate contained 20 plants (*n* = 20). Normal field management was implemented during the plant growth cycle. Seven samples (*n* = 7), each taken from an individual plant, were harvested from each replicate. The fruit samples were collected, frozen in liquid nitrogen, and kept in an ultra‐low‐temperature (−80°C) freezer for determination. Mix five samples from each repetition for transcriptome and metabolome analysis.

Transcriptome sequencing and data preprocessing were completed by Wuhan Boyuan Biotechnology Co., Ltd (Hubei, China). Total RNA was extracted from the outer epidermis of the tomato fruits from different treatment groups using the Polysaccharide and Polyphenol Plant RNA Extraction Kit (Huayueyang Biotechnology Co., Beijing, China) following its instructions. RNA‐seq libraries were constructed and sequenced on an Illumina HiSeq™ X‐Ten platform using the paired‐end mode (BGI, Shenzhen, China), with three biological replicates for each treatment. The raw sequencing data were assessed for quality and subjected to data filtering. Specifically, the reads with ambiguous nucleotides, low‐quality bases, and adapter sequences were filtered using the FastQC (version 0.11.5) and fastp (version 0.23.2) software with the default parameters (Chen et al., 2018). Then the clean reads from each library were mapped to the tomato genome (http://solgenomics.net, version SL2.5) using the hisat2 (version 2.2.1) software (Kim et al., 2019). Gene expression was determined based on the calculation of the fragments per kilobase of exon model per million mapped fragments (FPKM) of each gene in each sample using featureCounts (version 2.0.1) software (Love et al., 2014). Genes with a |log_2_ fold change| ≥1 and false discovery rate (FDR) <0.05 were identified as differentially expressed genes (DEGs) using DESeq2 package in R (Love et al., 2014). To understand the function of the DEGs, Gene Ontology (GO) enrichment analysis and Kyoto Encyclopedia of Genes and Genomes (KEGG) enrichment analysis were performed using clusterProfiler (version 4.2.2) software, with a corrected *P*‐value <0.05 were considered significantly enriched.

Metabolites analysis was performed following the standardized procedures established by Beijing Novogene Bioinformatics Technology Co., Ltd (Beijing, China). Metabolites were extracted from the freeze‐dried outer epidermis of the tomato fruits from different treatment groups using 80% cold aqueous methanol. The extracts of samples were analyzed using a Vanquish UHPLC system coupled with a Q Exactive™ HF‐X mass spectrometer (Thermo Fisher Scientific, MA, USA). The liquid chromatography (LC) conditions included a HypersilGold C18 column, a gradient of water with 0.1% acetic acid (A) and methanol (B), and a flow rate of 0.2 mL min^−1^. The mass spectrometry (MS) conditions included a scan range of 100–1500 m/z, a spray voltage of 3.5 kV, and a collision cell time of 30 ms. Metabolomics data were processed as previously described (Zhao et al., 2024). Metabolites were identified by matching MS/MS spectra with reference spectra in the KEGG (https://www.genome.jp/kegg/pathway.htm), LIPIDMaps (http://www.lipidmaps.org/), and HMDB (https://hmdb.ca/metabolites) databases. The metabolite quantification and analysis were performed using metaX software (Wen et al., 2017). The differentially accumulated metabolites (DAMs) were identified by partial least squares‐discriminant analysis (PLS‐DA). Metabolites satisfying |log_2_ fold change| ≥1 and variable importance of the projection (VIP) ≥1 were defined as DAMs at *P*‐value <0.05. Analysis on GO enrichment and KEGG enrichment were performed using the same approach as that in transcriptome.

Moreover, the nine‐quadrant correlation diagram was plotted using R, and a joint pathway analysis of the transcriptome and metabolome was conducted to screen common enriched KEGG pathways and the corresponding DEGs and DAMs according to the pathway annotation.

## AUTHOR CONTRIBUTIONS

Conceptualization, XW; methodology, XW, YC, WG; investigation, YC, WG, YZ, SQ, ZQ, CD, ZL, YD, JL, ZH, JL, XL, LL; data curation, XW, YC, WG; writing original draft preparation, YC, WG; writing—review and editing, YC, WG, XW; supervision, XW; funding acquisition, XW, YC, WG. All authors have read and agreed to the published version of the manuscript.

## CONFLICT OF INTEREST

The authors declare that they have no competing interests.

## Supporting information


**Figure S1.** Nucleotide sequences alignment of CDS between Sopen03g034650 and Solyc03g115660.
**Figure S2.** Protein sequences alignment between Sopen03g034650 and Solyc03g115660.
**Figure S3.** Nucleotide sequences alignment of CDS between Sopen03g034660 and Solyc03g115680.
**Figure S4.** Protein sequences alignment between Sopen03g034660 and Solyc03g115680.
**Figure S5.** The predicted signal peptides in the protein coded by the Cr3a gene.
**Figure S6.** The predicted transmembrane domains on the protein coded by the Cr3a gene.
**Figure S7.** Prediction of protein secondary structure of *Sopen03g034650*.
**Figure S8.** Prediction of protein secondary structure of *Solyc03g115660*.
**Figure S9.** The templates were found to match the target sequence.
**Figure S10.** The cysteine and methionine metabolism pathway plot identified by the integrated analysis of differentially expressed genes and differential accumulated metabolites from transcriptomic and metabolomic profiling at the mature green stage (blue dot represents downregulated).
**Figure S11.** The phenylpropanoid biosynthesis pathway plot identified by the integrated analysis of differentially expressed genes and differential accumulated metabolites from transcriptomic and metabolomic profiling at the mature green stage (red dot represents upregulated; blue dot represents downregulated).
**Figure S12.** The phenylalanine, tyrosine and tryptophan biosynthesis pathway plot identified by the integrated analysis of differentially expressed genes and differential accumulated metabolites from transcriptomic and metabolomic profiling at the red ripe stage (red dot represents upregulated; blue dot represents downregulated).
**Figure S13.** The phenylpropanoid biosynthesis pathway plot identified by the integrated analysis of differentially expressed genes and differential accumulated metabolites from transcriptomic and metabolomic profiling at the red ripe stage (red dot represents upregulated; blue dot represents downregulated).
**Figure S14.** The linoleic acid metabolism pathway plot identified by the integrated analysis of differentially expressed genes and differential accumulated metabolites from transcriptomic and metabolomic profiling at the red ripe stage (blue dot represents downregulated).

**Table S1.** The major InDel molecular markers developed in this study for target gene fine‐mapping.
**Table S2.** (A) The primers used in the real‐time PCR in this experiment. (B) The primers used in the transgenic experiment for PCR reaction of vector construction. (C) The primers used in the transgenic experiment for colony PCR identification after vector construction.
**Table S3.** Expression profile obtained based on RNA‐seq.
**Table S4.** Metabolome obtained based on LC‐MS.

## Data Availability

The that support the findings of this study and materials used in this study are available from the corresponding author upon reasonable request. The sequences obtained were deposited in the GenBank Short Read Archive (Accession SRP544696).
