# Supplementary material for: Cr3a, a candidate gene conferring fruit cracking resistance, was fine‐mapped in an introgression line of Solanum lycopersicum L
Source: Plant J. 2025 May 2;122(2):e70184. doi: 10.1111/tpj.70184 (PMC12047205; doi:10.1111/tpj.70184)

**Figure S1** Nucleotide sequences alignment of CDS between Sopen03g034650 and Solyc03g115660

CLUSTAL O(1.2.4) multiple sequence alignment

Sopen03g034650 ATGGGTTGTGTGGCGTCCAAGCAGACTGTGTCGGTTACTCCTGCATTTGATCATTCGGGG 60

Solyc03g115660 ATGGGTTGTGTGGCGTCCAAGCAGACTGTGTCGGTTACTCCTGCATTTGATCATTCGGGG 60

************************************************************

Sopen03g034650 ATTCTCGTTAGAGATGGGGAAGATGTCGGGTCAGGTAGGGGTCGGGTTGGGAGTGGTGGG 120

Solyc03g115660 ATTCTCGTTAGAGATGGGGAAGATGTCGGGTCGGGTAGGGGTCGGGTTGGGAGTGGTGGG 120

******************************** ***************************

Sopen03g034650 TTTGGGTTGGATTTTGATTTGAAGAAGGTGAAGAAGAGGGGAGATTCGGGTCTGAGTGGT 180

Solyc03g115660 TTTGGGTTGGATTTTGATTTGAAGAAGGTGAAGAAGAGGGGAGATTCGGGT--------- 171

***************************************************

Sopen03g034650 GCGAGGAGCGAGTTGGATGAGTCAGGTAGGGCAAGTTCGAATGGGTGTGGTAGTGAGTCA 240

Solyc03g115660 GCGGGGAGCGAGTTGGATGAGTCAGGTAGGGCGAGTTCGAATGGGTGTGGTAGTGAGTCA 231

*** **************************** ***************************

Sopen03g034650 GTGAGTTTTAGGTTGGGGAATTTGCAGAAATATGTGGAAGGAGAACAAGTGGCTGCTGGG 300

Solyc03g115660 GTGAGTTTTAGGTTGGGGAATTTGCAGAAATATGTGGAAGGAGAACAAGTGGCTGCTGGG 291

************************************************************

Sopen03g034650 TGGCCTGCTTGGCTTAGTGCTGTAGCAGGGGAAGCCATTCAAGGATGGGTCCCTCTCAGA 360

Solyc03g115660 TGGCCTGCTTGGCTTAGTGCTGTAGCAGGGGAAGCCATTCAAGGATGGGTGCCTCTCAGA 351

************************************************** *********

Sopen03g034650 GCTGAGTCTTTCGAAAAATTGGAAAAGATAGGTCAGGGTACATACAGCAGCGTATTCAGA 420

Solyc03g115660 GCTGAGTCCTTCGAAAAATTGGAAAAGATAGGTCAGGGTACATACAGCAGCGTATTCAGA 411

******** ***************************************************

Sopen03g034650 GCACGTGATTTAGAAAGTGGAAGGACAGTTGCCCTGAAGAAGGTGCGGTTTGATAACTTC 480

Solyc03g115660 GCACGTGATTTAGAAAGTGGAAGAACAGTTGCCCTGAAGAAGGTGCGGTTTGATAACTTC 471

*********************** ************************************

Sopen03g034650 GAGCCAGAAAGTGTTAGATTTATGGCACGAGAAATTATGATCCTCCGCAGGCTTGATCAC 540

Solyc03g115660 GAGCCAGAAAGTGTTAGATTTATGGCACGAGAAATTATGATCCTCCGCAGGCTTGATCAC 531

************************************************************

Sopen03g034650 CCCAATATCATCAAATTAGAAGGTCTAATTACCTCCAGATTGTCTTGTAGCATGTATCTT 600

Solyc03g115660 CCCAATATCATCAAATTAGAAGGTCTAATTACCTCCAGATTGTCTTGTAGCATGTATCTT 591

************************************************************

Sopen03g034650 GTGTTCGAGTATATGGAACATGATATTTCAGGACTCCTGTCTTGTCCAGAAGTTGAGTTC 660

Solyc03g115660 GTGTTCGAGTATATGGAACATGATATTTCAGGACTCCTGTCTTGTCCAGAAGTTGAGTTC 651

************************************************************

Sopen03g034650 AGCGAATCACAGATTAAATGCTACATGAAGCAGTTGTTGTCTGGAATCGAGCATTGTCAT 720

Solyc03g115660 AGCGAATCACAGATTAAATGCTACATGAAGCAGTTGTTGTCTGGAATCGAGCATTGTCAT 711

************************************************************

Sopen03g034650 TCTCGAGGTGTAATGCATCGGGACATCAAAGGTGCAAATCTTCTGGTAAATAATGACGGA 780

Solyc03g115660 TCTCGAGGTGTAATGCATCGGGACATCAAAGGTGCAAATCTTCTGGTAAATAATGACGGA 771

************************************************************

Sopen03g034650 ATTTTAAAGGTGGCAGATTTTGGATTGGCGAACTTCTGTAACCTTGGAAGGAAGCAACCA 840

Solyc03g115660 ATTTTAAAGGTGGCAGATTTTGGATTGGCGAACTTCTGTAACCTTGGAAGGAAGCAACCA 831

************************************************************

Sopen03g034650 CTAACCAGCCGAGTTGTCACTTTATGGTATCGTCCTCCAGAGCTTCTGTTGGGCTCCACA 900

Solyc03g115660 CTAACCAGCCGAGTTGTCACTTTATGGTATCGTCCTCCAGAGCTTCTGTTGGGCTCCACA 891

************************************************************

Sopen03g034650 GAGTATGGAGCCTCTGTGGATCTTTGGAGTGTCGGATGCGTGTTTGGTGAACTTCTTACT 960

Solyc03g115660 GAGTATGGAGCCTCTGTGGATCTTTGGAGTGTCGGATGCGTGTTTGGTGAACTTCTTACT 951

************************************************************

Sopen03g034650 GGTAAACCCATTCTTCAGGGGAGAACTGAGGTTGAGCAGCTGCATAAAATTTTTAAGCTC 1020

Solyc03g115660 GGTAAACCCATTCTTCAGGGGAGAACTGAGGTTGAACAGCTGCATAAAATTTTTAAGCTC 1011

*********************************** ************************

Sopen03g034650 TGTGGATCCCCACCTGATGATTACTGGAAGAAATCTAAACTTCCTCATGCAACTCTATTT 1080

Solyc03g115660 TGTGGATCCCCACCAGATGATTACTGGAAGAAATCTAAACTTCCTCATGCAACTCTATTT 1071

************** *********************************************

Sopen03g034650 AAACCGCAACATCCTTATGAGAGCTGTCTGTGGGATACATTTAAAGATCTAACCAAAAAT 1140

Solyc03g115660 AAACCACAACATCCTTATGAGAGCTGTCTGTGGGATACATTTAAAGATCTAACCAAAAGT 1131

***** **************************************************** *

Sopen03g034650 TCAGTCTCACTCATAGAGACTCTTCTTTCAGTGGAGCCACCGAAACGTGGAACTGCTTCT 1200

Solyc03g115660 TCAGTCTCACTCATAGAGACTCTTCTTTCAGTGGAGCCACCGAAACGTGGAACTGCTTCT 1191

************************************************************

Sopen03g034650 TCTGCCCTTGCATCTGAGTACTTCAAGACAAAGCCTCATGCCTGCGATCCTTTAAGCCTG 1260

Solyc03g115660 TCTGCCCTTGCATCTGAGTACTTCAAGACAAAGCCTCATGCCTGCGATCCTTTAAGCCTG 1251

************************************************************

Sopen03g034650 CCAAAGTATCCACCAAGCAAAGAGATTGATGCCAAACACTGTGAAGAAGCAAAAAGGAAG 1320

Solyc03g115660 CCAAAGTATCCACCAAGCAAAGAGATTGATGCCAAACACTGTGAAGAAGCAAAAAGGAAG 1311

************************************************************

Sopen03g034650 AAGCCTAGTGGAAGAGCCCGTGGACCTGAGACAACCAGAAAATCAATCAGAAAACAGAAT 1380

Solyc03g115660 AAGCCTAGTGGAAGAGCCCGTGGACCTGAGACAACCAGAAAGTCAATCAGAAAACAGAAT 1371

***************************************** ******************

Sopen03g034650 GCGACAAATAAACTGGCACCTGAAGAGAAGTTACCTGTCCAAAATCAAGGTGTTCCTAAA 1440

Solyc03g115660 GCGACAAATAAACTGGCACCTGAAGAGAAGTTACCTGTCCAAAATCAAGGTGTTCCTAAA 1431

************************************************************

Sopen03g034650 AGTAATGGTAGTAGTCTGGGTACTCTAAAAGAAGGAGACATTATTATAGGTCTTGAGCGA 1500

Solyc03g115660 AGTAATGGTAGTAGTCTGGGTACTCTAAAAGAAGGAGACATTATTATAGGTCTTGAGCGA 1491

************************************************************

Sopen03g034650 CCTAAGCCATCTGTCGATTTTATGGGAGAGGCTTCCCACATTAAGAATGCGTCTCAAGGC 1560

Solyc03g115660 CCTAAGCCATCTGTCGATTTTAGGGGAGAGGCTTCCCACATTAAGAATGCATCTCAAGGC 1551

********************** *************************** *********

Sopen03g034650 GATGTCCCCTTTTCAGGCCCTCTTCAAGTTTCAGGATCAAGTGGTTTTGCATGGGCGAAA 1620

Solyc03g115660 GATGTCCCCTTTTCAGGCCCTCTTCAAGTTTCAGGATCAAGTGGTTTTGCATGGGCGAAA 1611

************************************************************

Sopen03g034650 AGACGGGTTGATGATTCGTCAATGAGATCAAGGAGTAAATCAAGTTCAAGAAGCCTAAAG 1680

Solyc03g115660 AGACGGGTTGATGATTCGTCAATGAGATCAAGGAGTAAATCAAGTTCAAGAAGCCTAAAG 1671

************************************************************

Sopen03g034650 TTTGAACCTTCAGGTGCAGTTCATACTAAAAATAACACGGAGCTAAAAAAACAAGAGAAC 1740

Solyc03g115660 TTTGAACCTTCAGGTGCAGTTCATACTAAAAATAACACGGAGCTAAAAAAACAAGAGAAC 1731

************************************************************

Sopen03g034650 TATGAAGCTACAAATGGAAGTCGCACCAATTCTAAGGGCCGTGACACCTATGAATCCACC 1800

Solyc03g115660 TATGTAGCTACAAATGGAAGTCACACCAATTCTAAGGGCCGTGACACCTATGAATCCACC 1791

**** ***************** *************************************

Sopen03g034650 AAACATGCAATGCAACGACATTGGAGCCAATTAGAGCAGACAGATTCATTTGATGCTTCT 1860

Solyc03g115660 AAACATGCAATGCAACGACATTGGAGCCAATTAGAGCAGACAGATTCATTTGATACTTCT 1851

****************************************************** *****

Sopen03g034650 GATGGCTATCATTCTCAAGAACTGTCACTGGCTCTCTATCTGAAAGAGGAGACAGCTTTC 1920

Solyc03g115660 GATGGCTATCATTCTCAAGAACTGTCACTGGCTCTCTATCTGAAAGAGGAGACAGCTTTC 1911

************************************************************

Sopen03g034650 AAGAGGATCAACGTGGTTCAGGATCAAATGGACAAAGTTGAATTCTCAGGACCTTTGCTA 1980

Solyc03g115660 AAGAGGATCAATGTGGTTCAGGATCAAATGGACAAAGTTGAATTCTCAGGACCTTTGCTA 1971

*********** ************************************************

Sopen03g034650 TCTCAATCACACAGAGTTGAAGAACTCCTGGAGAAACATGAGCGCCAGATCCGACAAGCT 2040

Solyc03g115660 TCTCAATCACACAGAGTTGAAGAACTCCTGGAGAAACATGAGCGCCAGATCCGACAAGCT 2031

************************************************************

Sopen03g034650 GTTAGACGATCATGGTTCCAAAGAGTGAGGAAAAATGGGAACTGA--------------- 2085

Solyc03g115660 GTTAGACGATCATGGTTCCAAAGAGTGAGGAAAAACGGAAACTGATTGTATGGTGCTCCT 2091

*********************************** ** ******

Sopen03g034650 ------------------------------------------------------------ 2085

Solyc03g115660 ATTTCACACGGTATACTATACAGTAAAGAAACGAGGTACAGGAATTTTGACAGTGGATTC 2151

Sopen03g034650 ------------------------------------------------------------ 2085

Solyc03g115660 ACAACAATCAAGAAAGTCTCACATACTCTAAAGTAGCTGAACGTGATGAGCTGTTGATCT 2211

Sopen03g034650 ---------------------------------------- 2085

Solyc03g115660 CGATCCATAGCAGTGTGCAGAAGACTCACAAAGTTAAGAA 2251

**Figure S2** Protein sequences alignment between Sopen03g034650 and Solyc03g115660

**
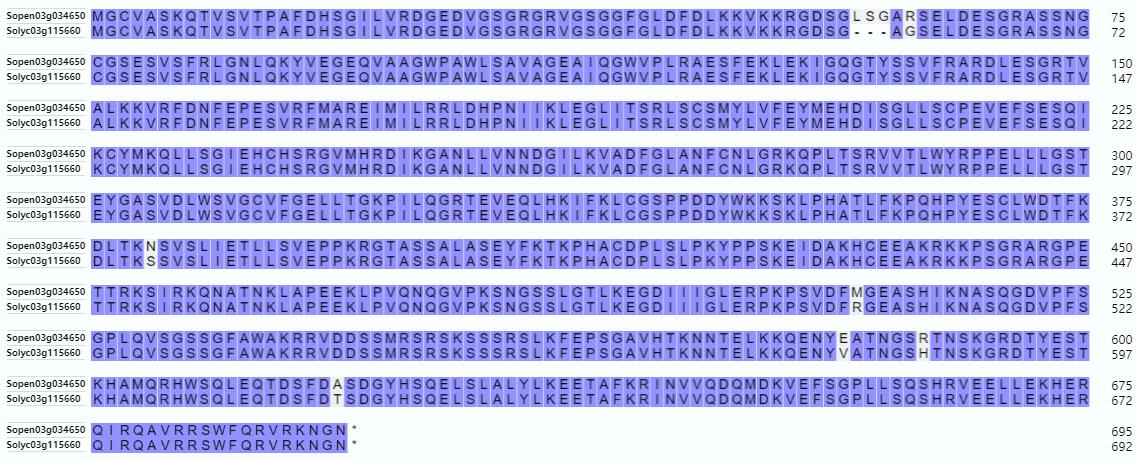
**

**Figure S3** Nucleotide sequences alignment of CDS between Sopen03g034660 and Solyc03g115680

CLUSTAL O(1.2.4) multiple sequence alignment

Sopen03g034660 ------------------------------------------------------------ 0

Solyc03g115680 ATGGGTTGTGTGGCGTCCAAGCAGACTGTGTCGGTTACTCCTGCATTTGATCATTCGGGG 60

Sopen03g034660 ------------------------------------------------------------ 0

Solyc03g115680 ATTCTCAAGGTGAAGAAGAGGGGAGATTCGGGTGCGGGGAGCGAGTTGGATGAGTCAGGT 120

Sopen03g034660 ------------------------------------------------------------ 0

Solyc03g115680 AGGGCGAGTTCGAATGGGTGTGGTAGTGAGTCAGTGAGTTTTAGGTTGGGGAATTTGCAG 180

Sopen03g034660 ------------------------------------------------------------ 0

Solyc03g115680 AAATATGTGGAAGGAGAACAAGTGGCTGCTGGGTGGCCTGCTTGGCTTAGTGCTGTAGCA 240

Sopen03g034660 ------------------------------------------------------------ 0

Solyc03g115680 GGGGAAGCCATTCAAGGATGGGTGCCTCTCAGAGCTGAGTCCTTCGAAAAATTGGAAAAG 300

Sopen03g034660 ------------------------------------------------------------ 0

Solyc03g115680 CTTAGGGATGTCTCAAAAGTACTTTCTCATTTGATTGTGATATATCAGGTTAACGTCTCA 360

Sopen03g034660 ------------------------------------------------------------ 0

Solyc03g115680 TATGGCAGGATAGGTCAGGGTACATACAGCAGCGTATTCAGAGCACGTGATTTAGAAAGT 420

Sopen03g034660 ------------------------------------------------------------ 0

Solyc03g115680 GGAAGAACAGTTGCCCTGAAGAAGGTGCGGTTTGATAACTTCGAGCCAGAAAGTGTTAGA 480

Sopen03g034660 ------------------------------------------------------------ 0

Solyc03g115680 TTTATGGCACGAGAAATTATGATCCTCCGCAGGCTTGATCACCCCAATATCATCAAATTA 540

Sopen03g034660 ------------------------------------------------------------ 0

Solyc03g115680 GAAGGTCTAATTACCTCCAGATTGTCTTGTAGCATGTATCTTGTGTTCGAGTATATGGAA 600

Sopen03g034660 ------------------------------------------------------------ 0

Solyc03g115680 CATGATATTTCAGGACTCCTGTCTTGTCCAGAAGTTGAGTTCAGCGAATCACAGATTAAA 660

Sopen03g034660 ------------------------------------------------------------ 0

Solyc03g115680 TGCTACATGAAGCAGTTGTTGTCTGGAATCGAGCATTGTCATTCTCGAGGTGTAATGCAT 720

Sopen03g034660 ------------------------------------------------------------ 0

Solyc03g115680 CGGGACATCAAAGGTGCAAATCTTCTGGTAAATAATGACGGAATTTTAAAGGTGGCAGAT 780

Sopen03g034660 ------------------------------------------------------------ 0

Solyc03g115680 TTTGGATTGGCGAACTTCTGTAACCTTGGAAGGAAGCAACCACTAACCAGCCGAGTTGTC 840

Sopen03g034660 ------------------------------------------------------------ 0

Solyc03g115680 ACTTTATGGTATCGTCCTCCAGAGCTTCTGTTGGGCTCCACAGAGTATGGAGCCTCTGTG 900

Sopen03g034660 ------------------------------------------------------------ 0

Solyc03g115680 GATCTTTGGAGTGTCGGATGCGTGTTTGGTGAACTTCTTACTGGTAAACCCATTCTTCAG 960

Sopen03g034660 ------------------------------------------------------------ 0

Solyc03g115680 GGGAGAACTGAGGTTGAACAGCTGCATAAAATTTTTAAGCTCTGTGGATCCCCACCAGAT 1020

Sopen03g034660 ------------------------------------------------------------ 0

Solyc03g115680 GATTACTGGAAGAAATCTAAACTTCCTCATGCAACTCTATTTAAACCACAACATCCTTAT 1080

Sopen03g034660 ------------------------------------------------------------ 0

Solyc03g115680 GAGAGCTGTCTGTGGGATACATTTAAAGATCTAACCAAAAGTTCAGTCTCACTCATAGAG 1140

Sopen03g034660 ------------------------------------------------------------ 0

Solyc03g115680 ACTCTTCTTTCAGTGGAGCCACCGAAACGTGGAACTGCTTCTTCTGCCCTTGCATCTGAG 1200

Sopen03g034660 ------------------------------------------------------------ 0

Solyc03g115680 TACTTCAAGACAAAGCCTCATGCCTGCGATCCTTTAAGCCTGCCAAAGTATCCACCAAGC 1260

Sopen03g034660 ------------------------------------------------------------ 0

Solyc03g115680 AAAGAGATTGATGCCAAACACTGTGAAGAAGCAAAAAGGAAGAAGCCTAGTGGAAGAGCC 1320

Sopen03g034660 ------------------------------------------------------------ 0

Solyc03g115680 CGTGGACCTGAGACAACCAGAAAGTCAATCAGAAAACAGAATGCGACAAATAAACTGGCA 1380

Sopen03g034660 ------------------------------------------------------------ 0

Solyc03g115680 CCTGAAGAGAAGTTACCTGTCCAAAATCAAGGTGTTCCTAAAAGTAATGGTAGTAGTCTG 1440

Sopen03g034660 ------------------------------------------------------------ 0

Solyc03g115680 GGTACTCTAAAAGAAGGAGACATTATTATAGGTCTTGAGCGACCTAAGCCATCTGTCGAT 1500

Sopen03g034660 ------------------------------------------------------------ 0

Solyc03g115680 TTTAGGGGAGAGGCTTCCCACATTAAGAATGCATCTCAAGGCGATGTCCCCTTTTCAGGC 1560

Sopen03g034660 ------------------------------------------------------------ 0

Solyc03g115680 CCTCTTCAAGTTTCAGGATCAAGTGGTTTTGCATGGGCGAAAAGACGGGTTGATGATTCG 1620

Sopen03g034660 ------------------------------------------------------------ 0

Solyc03g115680 TCAATGAGATCAAGGAGTAAATCAAGTTCAAGAAGCCTAAAGTTTGAACCTTCAGGTGCA 1680

Sopen03g034660 ------------------------------------------------------------ 0

Solyc03g115680 GTTCATACTAAAAATAACACGGAGCTAAAAAAACAAGAGAACTATGTAGCTACAAATGGA 1740

Sopen03g034660 ------------------------------------------------------------ 0

Solyc03g115680 AGTCACACCAATTCTAAGGGCCGTGACACCTATGAATCCACCAAACATGCAATGCAACGA 1800

Sopen03g034660 ------------------------------------------------------------ 0

Solyc03g115680 CATTGGAGCCAATTAGAGCAGACAGATTCATTTGATACTTCTGATGGCTATCATTCTCAA 1860

Sopen03g034660 ------------------------------------------------------------ 0

Solyc03g115680 GAACTGTCACTGGCTCTCTATCTGAAAGAGGAGACAGCTTTCAAGAGGATCAATGTGGTT 1920

Sopen03g034660 -----------------------------------------------ATG--GCGCTGTT 11

Solyc03g115680 CAGGATCAAATGGACAAAGTTGAATTCTCAGGACCTTTGCTATCTCAATCACACAGAGTT 1980

** * ***

Sopen03g034660 CAGACGGTTC-----TTCTATAGGAAGCCGCCGGATCGGCTTTTAGAGATCTCTGAGCGG 66

Solyc03g115680 GAAGAACTCCTGGAGAAACATGAGCGCCAGATCCGACAAGCTGTTAGACGATCATGGTTC 2040

* * * ** * * * * * * ** *

Sopen03g034660 GTTTACGTGTTTGATTGCTGCTTCTCCACTGACGTGTTGGATGAAGATGAGTACAAGACA 126

Solyc03g115680 CAAAGAGTGTTTGATTGCTGCTTCTCCACTGACGTGTTGGATGAAGATGAGTACAAGACA 2100

******************************************************

Sopen03g034660 TATATGGGGGGGATTGTAGCTCAGCTGCAGGACCACTATGCAGATGCTTCTTTCATGGTT 186

Solyc03g115680 TATATGGGGGGGATTGTAGCTCAGCTGCAGGACCACTATGCAGATGCTTCTTTCATGGTT 2160

************************************************************

Sopen03g034660 TTTAACTTTAGGGAAGGTGATAGGAGGAGCCAAATATCTGACATATTGTCTCAGTATGAT 246

Solyc03g115680 TTTAACTTTAGGGAAGGTGATAGGAGGAGCCAAATATCTGACATATTGTCTCAGTATGAT 2220

************************************************************

Sopen03g034660 ATGACTGTGATGGATTATCCTCGGCAATATGAAGGGTGTCCACTTCTGCCTCTGGAGATG 306

Solyc03g115680 ATGACTGTGATGGATTATCCTCGGCAATATGAAGGGTGTCCACTTCTGCCTCTGGAGATG 2280

************************************************************

Sopen03g034660 ATCCACCACTTCTTGCGCTCAAGTGAGAGTTGGCTTTCACTTGAGGGTCAGCAAAACGTC 366

Solyc03g115680 ATCCACCACTTCTTGCGCTCAAGTGAGAGTTGGCTTTCACTTGAGGGTCAGCAAAACGTC 2340

************************************************************

Sopen03g034660 CTGTTAATGCACTGTGAGAGGGGAGGCTGGCCTGTACTTGCTTTTATGCTTGCTGGCCTT 426

Solyc03g115680 CTGTTAATGCACTGTGAGAGGGGAGGCTGGCCTGTACTTGCTTTTATGCTTGCTGGCCTT 2400

************************************************************

Sopen03g034660 CTTTTGTACCGGAAACAGTACACTGGGGAGCTTAAAACTCTTGAAATGGTATACAAGCAG 486

Solyc03g115680 CTTTTGTACCGGAAACAGTACACTGGGGAGCTTAAAACTCTTGAAATGGTATACAAGCAG 2460

************************************************************

Sopen03g034660 GCCCCTAGGGAGCTGCTTCATCTTTTGTCTCCTCTGAATCCACAGCCATCTCAGCTTAGA 546

Solyc03g115680 GCCCCTAGGGAGCTGCTTCATCTTTTGTCTCCTCTGAATCCACAGCCATCTCAGCTTAGA 2520

************************************************************

Sopen03g034660 TATCTCCAGTACATCTCCAGAAAAAATTTTGGTTCAGAATGGCTGCCATCAGATACACCT 606

Solyc03g115680 TATCTCCAGTACATCTCCAGAAAAAATTTTGGTTCAGAATGGCTGCCATCAGATACACCT 2580

************************************************************

Sopen03g034660 TTTGCCTTAGATTGCATCAAACTTAGTTTTCCTCCTCTATTTGATGGAGGGAGAGGCTGT 666

Solyc03g115680 TTTGCCTTAGATTGCATTAAACTTAGTTTTCCTCCTCTATTTGATGGAGGGAGAGGCTGT 2640

***************** ******************************************

Sopen03g034660 CGACCTGTACTTCGCGTCTATGGACAGGACCCTGCTTCAACAACCTCTAATAGGAGCTCT 726

Solyc03g115680 CGACCTGTACTTCGCGTCTATGGACAGGACCCTGCTTCAACAACCTCTAATAGGAGCTCT 2700

************************************************************

Sopen03g034660 AAGCTCCTCTTTTCAACTCTAAAGACAAAAAGACATGCTCGCTTCTACCAACAGGAAGAG 786

Solyc03g115680 AAGCTCCTCTTTTCAACTCTAAAGACAAAAAAACATGCTCGCTTCTACCAACAGGAAGAG 2760

******************************* ****************************

Sopen03g034660 TGTTCAATGGTGAAAATTGACATCCATTGTCGTGTCCAAGGAGATGTTGTTCTCGAGTGT 846

Solyc03g115680 TGTTCAATGGTGAAAATTGACATCCATTGTCGTGTCCAAGGAGATGTTGTTCTCGAGTGT 2820

************************************************************

Sopen03g034660 GTCCATTTGGAAGATGATCTAGTAAGGGAAGAAATGATGTTCAGGGTCATGTTCCACACT 906

Solyc03g115680 GTCCATTTGGAAGATGATCTAG-------------------------------------- 2842

**********************

Sopen03g034660 ACATTTATTCGCTCAAATGTTTTGATGTTAATGCGTGATGATGTTGATGTCCTGTGGGAT 966

Solyc03g115680 ------------------------------------------------------------ 2842

Sopen03g034660 GCAAAAGACCAATTTCCTAGAGGGTTCAAAGCAGAGGTACTCTTTTCGGATCCTAATGCT 1026

Solyc03g115680 -------------------------------------TACTCTTTTCGGATCCTAATGCT 2865

***********************

Sopen03g034660 GTTCCATCTGTTGTCACTGAGGAAGTGCCAAGTGAGGATGAGAATGGGACTGAAGGTGCT 1086

Solyc03g115680 GTTCCATCTGTTGTCACTGAGGAAGTGCCAAGTGAGGATGAGAATGGGACTGAAGGTGCT 2925

************************************************************

Sopen03g034660 TCACCTGAGGAGTTTTTCGAAGTCGAAGAGATATTCAGCAATGCTGTTGATGGGCAGGAT 1146

Solyc03g115680 TCACCTGAGGAGTTTTTCGAAGTCGAAGAGATATTCAGCAATGCTGTTGATGGGCAGGAT 2985

************************************************************

Sopen03g034660 GGAAGGGGGGAATCTGGAGCCCACATTGTCAAGGAAAGTTTGCAGGATGATGATAGTATT 1206

Solyc03g115680 GGAAGGGGGGAATCTGGAGCCCACATTGTCAAGGAAAGTTTGCAGGATGATGATAGTATT 3045

************************************************************

Sopen03g034660 GAAATGATCTGGAAAGAGGAGGTGGAACATCATGCATTTCAAGATTGTGCATCAGACGAA 1266

Solyc03g115680 GAAATGATCTGGAAAGAGGAGGTGGAACATCATGCATTTCAAGATTGTGCATCAGACGAA 3105

************************************************************

Sopen03g034660 GTAAATCACAAGCAAGAAGGAAAGATGGATTCTAATCGCTCTGCATCAGAAAAAAATATT 1326

Solyc03g115680 GTAAATCACAAGCAAGAAGGAAAGATGGATTCTAATCGCTCTGCATCAGAAAAAAATATT 3165

************************************************************

Sopen03g034660 TTAGGAGAGAGAGACAACTCTATACCCTCAAAGGTTATAGTTTCCAATGGTACTAGCAAC 1386

Solyc03g115680 TTAGGAGAGAGAGACAACTCTATATCCTCAAAGGTTATAGTTTCCAATGGTACTAGCAAC 3225

************************ ***********************************

Sopen03g034660 ATGGAATCAGAACAGGTAATATCTGGGGATTGTGTTGCATCAGAAAATGGGGAACTAAAG 1446

Solyc03g115680 ATGGAATCAGAACAGGTAATATCTGGGGATTGTGTTGCATCAGAAAATGGGGAACTAAAG 3285

************************************************************

Sopen03g034660 CAAGATAAAGAAGACACTCTGAGACAGAAGAAGTTAGAGAGAGAAGGTTCGCATCAGAAG 1506

Solyc03g115680 CAAGATAAAGAAGACACTCTGAGACAGAAGAAGTTAGAGAGGGAAGGTTCGCATCAGAAG 3345

***************************************** ******************

Sopen03g034660 GTGAGTGCTGATACTAGTAAACAAAAAAGTGACAAGACAACCTCATCTCTAAAGAAACAG 1566

Solyc03g115680 GTGAGTGCTGATACTAGTAAACAAAAAAGTGACAAGACAACCTCATCTCTAAAGAAACAG 3405

************************************************************

Sopen03g034660 TCATTTTCCAACGCTAAACCAGCTGCTGATGGCGTTGGTCCGAAAAATAAATCTAAACAG 1626

Solyc03g115680 TCATTTTCCAACGCTAAACCAGCTGCCGATGGCGTTGGTCCGAAAAATAAATCTAAACAG 3465

************************** *********************************

Sopen03g034660 CAGGAAATCCAAGGCACCGTTTTACGACCGGCAAAGCCTAATGCAGTGTCCCGGTGGATC 1686

Solyc03g115680 CAGGAAATCCAAGGCACCGTTTTACGACCGGCAAAGCCTAATGCAGTGTCCCGGTGGATC 3525

************************************************************

Sopen03g034660 CCTCCAAACAAAGGTTCTTACACTAGTTCAATGCATGTATCGTATCCACCATCAAGGTAT 1746

Solyc03g115680 CCTCCAAACAAAGGTTCTTACACTAGTTCAATGCATGTATCGTATCCACCATCAAGGTAT 3585

************************************************************

Sopen03g034660 AACAGTGCTCCTCCTGTGCTTGCCCTAACCAAGGATTTTCAGTCTGGGGTTAAATCAAAG 1806

Solyc03g115680 AACAGTGCTCCTCCTGTGCTTGCCCTAACCAAGGATTTTCAGTCTGGGGTTAAATCAAAG 3645

************************************************************

Sopen03g034660 TCACCATCTCCTCAAGCTTCTTCAGAAGCTATAGCTTCTGCTGAAGCAGGTCGTGTTTCA 1866

Solyc03g115680 TCACCATCTCCTCAAGCTTCTTCAGAAGCTATAGCTTCTGCTGAAGCAGGTCGTGTTTCA 3705

************************************************************

Sopen03g034660 GGGAAAGACTCTTCATGTTCTGCATCGGGTACGTCAATTGTGGAGGCATCTGTTGCTACA 1926

Solyc03g115680 GAGAAAGACTCTTCATGTTCTGCATCGGGTATGTCAATTGTGGAGGCATCTGTTGCTACA 3765

* ***************************** ****************************

Sopen03g034660 ATATCTGCCCCAGAATCAGTTGAAAGCCAGGCCCTGAAGCTTCATCCATCTCCTCCAAGT 1986

Solyc03g115680 ATATCTGCCCCAGAATCAGTTGAAAGCCAGGCCCTGAAGCTTCATCCATCTCCTCCAAGT 3825

************************************************************

Sopen03g034660 CCGCTTCTTTCTCCATCTCCAATTACTTCATCTCATGAACCTTCTGATACTGAGGTAGCA 2046

Solyc03g115680 CCGCTTCTTTCTCCATCTCCAATTACTTCATCTCACGAACCTTCTGATACTGAGGTAGCA 3885

*********************************** ************************

Sopen03g034660 GGAACTAATTCACCATCAGCGACTTCAGCTTTGTCTTCACAGGGGAGTAATATTATGACA 2106

Solyc03g115680 GGAACTAATTCACCATCAGCGACTTCAGCTTTGTCTTCACAGGGGAGTGCCTTTTCTCCT 3945

************************************************ ** *

Sopen03g034660 TCTTCTTTGACTCAGCCGGACCGTTATTCTTC------ACCTGCTCTGCCCGAACCTCCC 2160

Solyc03g115680 CCCCCACCACCACTGCCTCCTCCACCTTTGCCCTCATCATCCAGTGTAATAAATGCTGGT 4005

* * * * *** * ** * * * * * * **

Sopen03g034660 TCATTTTCACCCC------------------------------CTCCACCTCCCCCACCC 2190

Solyc03g115680 AGGGTCTTGCCACCACCCCCTCCACCACCCCTTTGGACAGGAGCGCCAACTCCACCACCT 4065

* * ** * * *** **** *****

Sopen03g034660 CCTCCACCTCCACCATTTACTAGCTCGAGGATGACTCCGCT---------GGATGCCTTT 2241

Solyc03g115680 CCACCTCCCCCTCCTTTTTCTAGAGCGCCAACTCCACCACCTCCCCCTCCTCCTCCTTTT 4125

** ** ** ** ** *** **** ** * * ** * * * ***

Sopen03g034660 TCTCCTCCCCCACCAC------CACCGCCTCCTCCACCTTTGCCCTCATCATCCAGTGTA 2295

Solyc03g115680 TCTAGAGCGCCAACTCCACCACCTCCACCTCCCCCTCCTTTTTCTAGAGCGCCAACTCCA 4185

*** * *** * * * ** ***** ** ***** * * * * * * *

Sopen03g034660 ATAAATGCTGGTAGGGTCTTGCC------------ACCACCCCCTCCACCACCCCTTTGG 2343

Solyc03g115680 CCACCTCCCCCTCCTTTTTCTAGAGCTCCAACTCCACCACCACCACCACCGCCTCCTTCA 4245

* * * * * * ****** ** ***** ** * **

Sopen03g034660 ACGGGGTATGGGACT-----TCCTTAGAAGTAGTATC-TAGTTCATCGCCACCTCCACCT 2397

Solyc03g115680 CGTGGTTCTGTACCACCACCTCCTCCTCCGCTGCCTGGAGCTCCACCTCCACCTATGTGG 4305

** * ** * **** * * * * ** * ******

Sopen03g034660 CCACCTCCACCTCCTCC---TCCTCCTATATATGCA------------TCCATTTTACCA 2442

Solyc03g115680 GGGCCTCCACCTCCTCCACCTCCTCCTATGCGGGGGCCTCCACCTCCACCTCCACCACCT 4365

************** ********* * * ***

Sopen03g034660 AGATTG---GCTAGCTTTGGGGGTTCCACATCGCCGCC---ACCTCCTCCCCCTCC---- 2492

Solyc03g115680 GGAGCACCTCCTCCAATGCGGGGCCCTCCACCTCCTCCACCTCCTCCTGGAGGTGGCCCT 4425

** ** * **** * ** * ** ** ****** *

Sopen03g034660 --------CCCTCCTCCTATGCATACTGGCTATTCTCCTCCTCCACCACCTCTACCTCCT 2544

Solyc03g115680 CCTCCACCACCTCCTCCAATACGTGGAGCTCCACCGCCCCCTCCTCCTCCTGGAGGTCGT 4485

******** ** * * * * ** ***** ** *** * ** *

Sopen03g034660 ACTCTTGC-CACACGAATCCTACATACCTCGCAAGACGCGCACCTGGTCCACCTCCCCCA 2603

Solyc03g115680 GCACCTGGTCCACCTCCCCCACCTCCTCCGGGAGGACGCGCACCTGGTCCACCTCCCCCA 4545

* * ** * * ** * * * * **************************

Sopen03g034660 CCCCCTCCTGGAGGGCGTGCACCTGGTCCACCTCCCCCACCCCCTCCGGGAGGACGCGCA 2663

Solyc03g115680 CCCCCTCCTGGAGGGCGTGCACCTGGTCCACCTCCCCCACCCCCTCCGGGAGGACGCGCA 4605

************************************************************

Sopen03g034660 CCTGGTCCACCTCCTCCACCGGGAGCTCCAAGACCTCCTGGTGGTGGACCTCCCCCACCA 2723

Solyc03g115680 CCTGGTCCACCTCCTCCACCGGGAGCTCCAAGACCTCCTGGTGGTGGACCACCACCACCA 4665

************************************************** ** ******

Sopen03g034660 CCACCTTTTGGTTCTAAAGGACCTGCAGTTGGCAGAGGCCTTCCTGCTGGGAGAGGGCAA 2783

Solyc03g115680 CCACCTTTTGGTTCTAAAGGACCTGCAGTTGGCAGAGGCCTTCCTGCTGGGAGAGGGCAA 4725

************************************************************

Sopen03g034660 GGATTTTCACGCGCAGCTGGTGGTGTAG-------------------------------- 2811

Solyc03g115680 GGATTTTCACGCGCAGCTGGTGGTGTAGCCCCACGAAGATCTAACTTGAAGCCGTTGCAT 4785

****************************

Sopen03g034660 ------------------------------------------------------------ 2811

Solyc03g115680 TGGAGCAAGGTAACTAGGGCACTTCAAGGAAGCTTATGGGATGAACTACAAAGAAACGGA 4845

Sopen03g034660 ------------------------------------------------------------ 2811

Solyc03g115680 GAGACTCAATTGTCACCAGAATTCGATTTTTCAGAACTTGAGACTCTTTTCTCTGCTACA 4905

Sopen03g034660 ------------------------------------------------------------ 2811

Solyc03g115680 GTCCCCAAGTCAGATAATGCGGGTAAATCTGGAGGGAGAAGGAAGTCTGTTGGGTCTAAA 4965

Sopen03g034660 ------------------------------------------------------------ 2811

Solyc03g115680 CCTGACAGGGTTCACCTGGTTGACTTGAGGAGGGCAAATAATACTGAAATTATGCTCACA 5025

Sopen03g034660 ------------------------------------------------------------ 2811

Solyc03g115680 AAGGTGAAAATGCCACTGCCTGACATGATGGCCGCTGCCCTTGCAATGGATGAGTCAATT 5085

Sopen03g034660 ------------------------------------------------------------ 2811

Solyc03g115680 TTAGATGCTGATCAGGTGGAGAATCTTATCAAGTTTTGTCCTACCAAAGATGAGATGGAA 5145

Sopen03g034660 ------------------------------------------------------------ 2811

Solyc03g115680 CTTCTCAAGAATTACACAGGTGACCAGGATCTTCTGGGAAAGTGTGAACAGTTTTTTCTG 5205

Sopen03g034660 ------------------------------------------------------------ 2811

Solyc03g115680 GAGCTCATGAAGGTGCCCCGAGTAGAGTCAAAACTAAGAGTTTTTCTCTTCAAGATCCAG 5265

Sopen03g034660 ------------------------------------------------------------ 2811

Solyc03g115680 TTCAACTCTCAGGTCACGGACTTCAAAAAAAGCTTGAACACAGTGAACTCTGCTTGTGAA 5325

Sopen03g034660 ------------------------------------------------------------ 2811

Solyc03g115680 GAGGTCCGACATTCTCTTAAATTGAAGGAAATATTGAAGAAAATATTGTATCTAGGGAAT 5385

Sopen03g034660 ------------------------------------------------------------ 2811

Solyc03g115680 GCATTGAACCAGGGAACTGCCAGAGGTTCTGCCATTGGATTTAAGTTGGACAGTCTTTTG 5445

Sopen03g034660 ------------------------------------------------------------ 2811

Solyc03g115680 AAGCTCACTGATACTCGTGCTACTAACAACAAGATGACACTCATGCATTATCTTTGTAAG 5505

Sopen03g034660 ------------------------------------------------------------ 2811

Solyc03g115680 GTTCTTGCTTCCAAGTCACCATCACTTTTAGACTTTCATGTAGATCTTGTAAGCCTGGAA 5565

Sopen03g034660 ------------------------------------------------------------ 2811

Solyc03g115680 GCTGCGTCAAAGATACAATTGAAGTCTTTGGCTGAAGAAATGCAAGCAATCATCAAGGGT 5625

Sopen03g034660 ------------------------------------------------------------ 2811

Solyc03g115680 TTGGAAAAAGTTAAAAAAGAACTGGAGGCTTCGGAGACTGATGGCCCTGTGTCTGAAATT 5685

Sopen03g034660 ------------------------------------------------------------ 2811

Solyc03g115680 TTTCGCAAGACCTTAAAGGAATTCGTTGGTGTGGCTGAAGCACAGGTTGGCTCTGTCAAG 5745

Sopen03g034660 ------------------------------------------------------------ 2811

Solyc03g115680 GATTTATATTCTGTTGCGGGCAGAAATGCAGATGCACTTGCACTATATTTCGGTGAGGAT 5805

Sopen03g034660 ------------------------------------------------------------ 2811

Solyc03g115680 CCTGCCCGTTGTCCGTTTGAGCAAGTCACGGCGACCCTCTTAAATTTTGTAAGGCTGTTC 5865

Sopen03g034660 ------------------------------------------------------------ 2811

Solyc03g115680 CGCAAAGCCCACGAAGAGAACTTGAAGCAGGCTGAATTAGAAAGGAAGAAAGTTCAAAAG 5925

Sopen03g034660 ------------------------------------------------------ 2811

Solyc03g115680 GAAGAAATAGAGAACGCCAAAGGAGTTAATCTCAATAAGAAGGGTTTCAAGTGA 5979

**Figure S4** Protein sequences alignment between Sopen03g034660 and Solyc03g115680

**
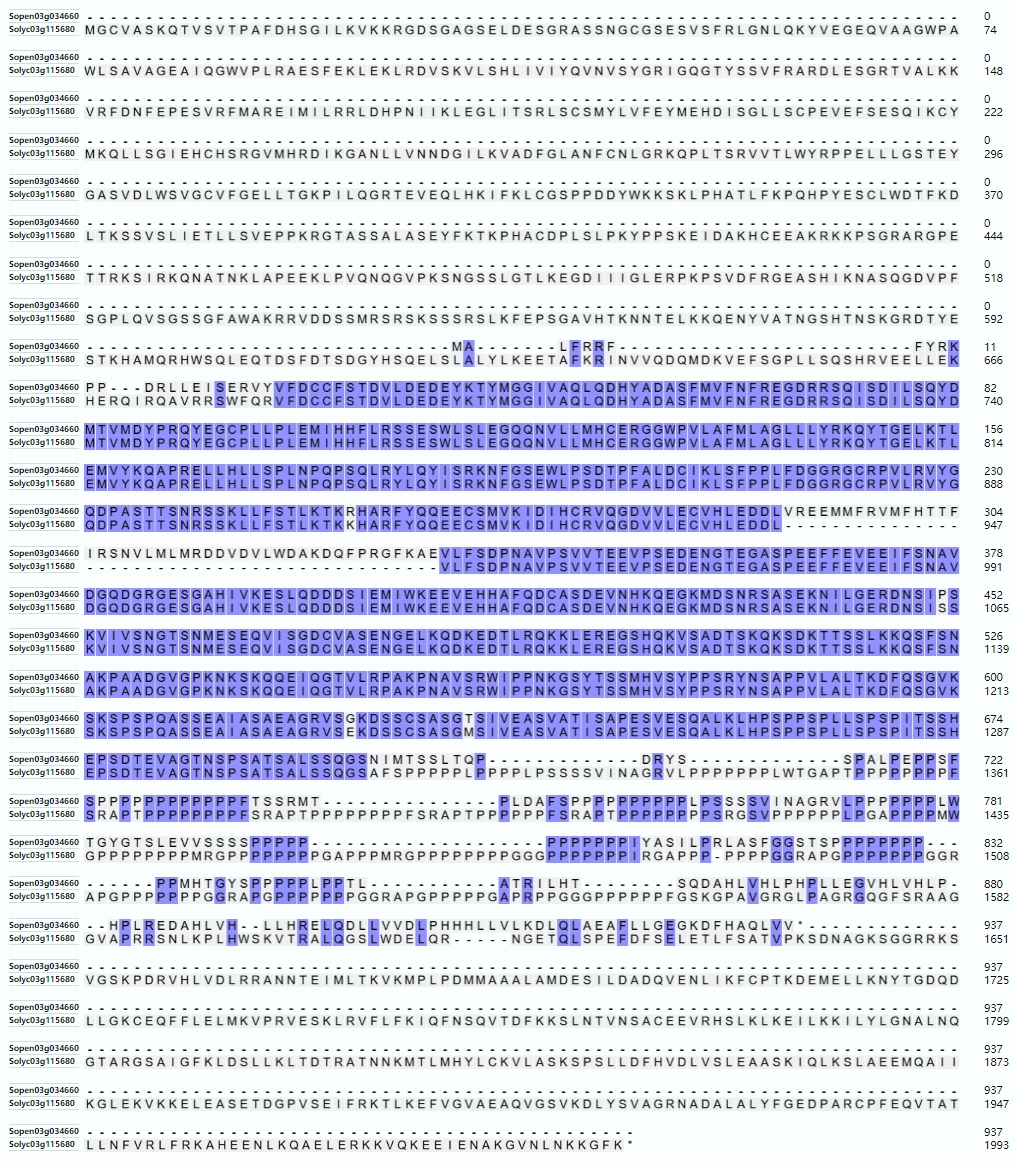
**

**Figure S5** The predicted signal peptides in the protein coded by the Cr3a gene.

**
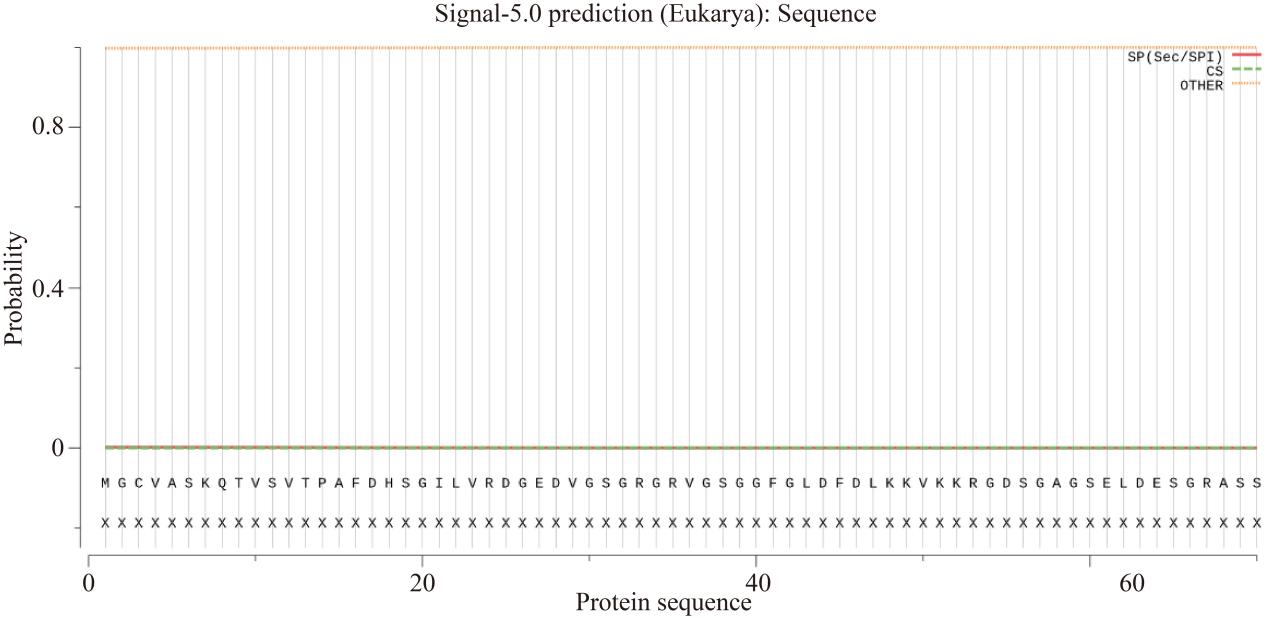
**

**Figure S6** The predicted transmembrane domains on the protein coded by the Cr3a gene.

**
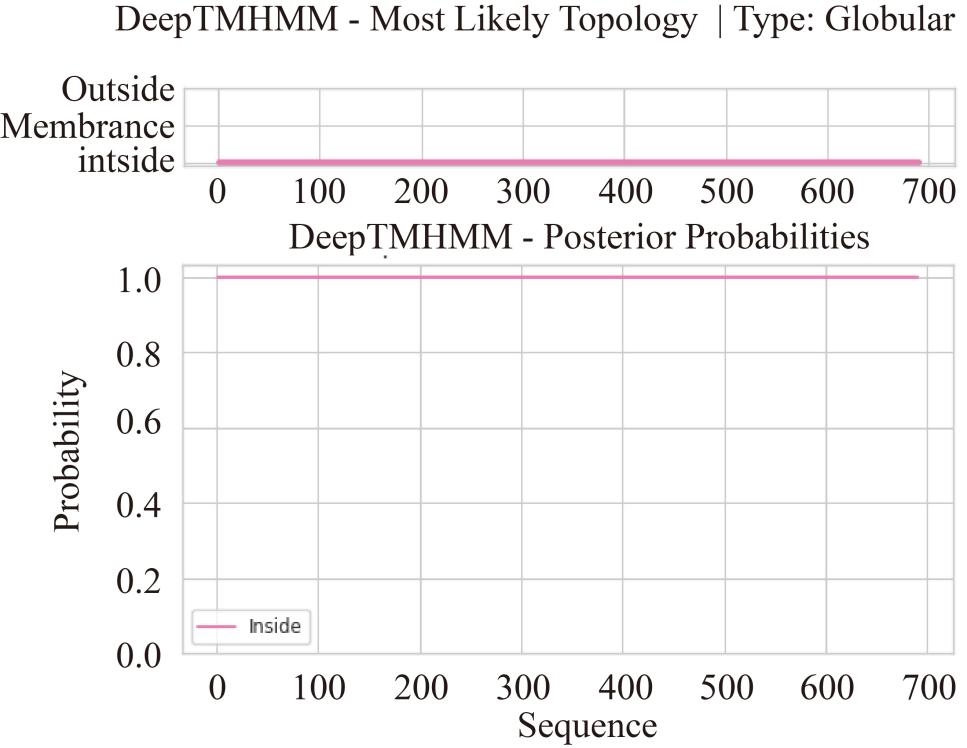
**

**Figure S7** Prediction of protein secondary structure of *Sopen03g034650*

**
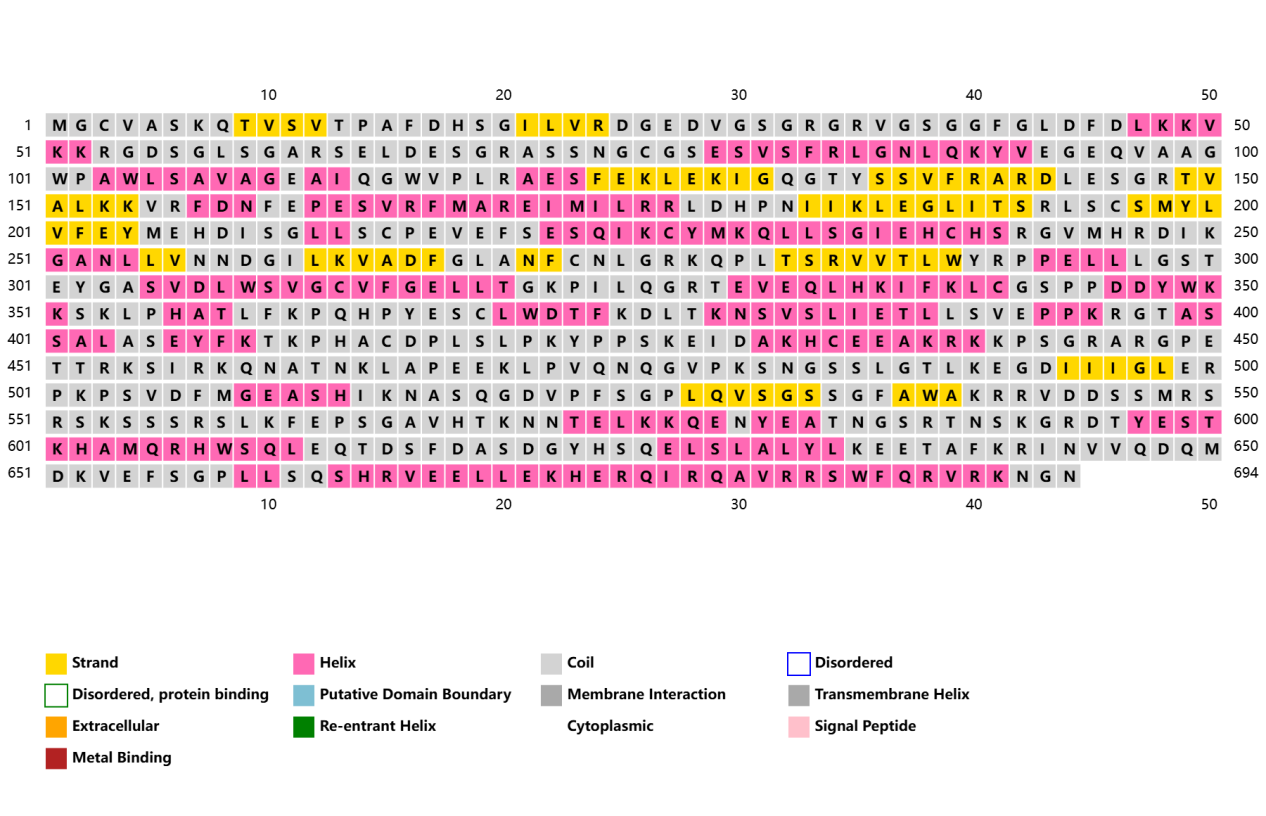
**

**Figure S8** Prediction of protein secondary structure of *Solyc03g115660*

**
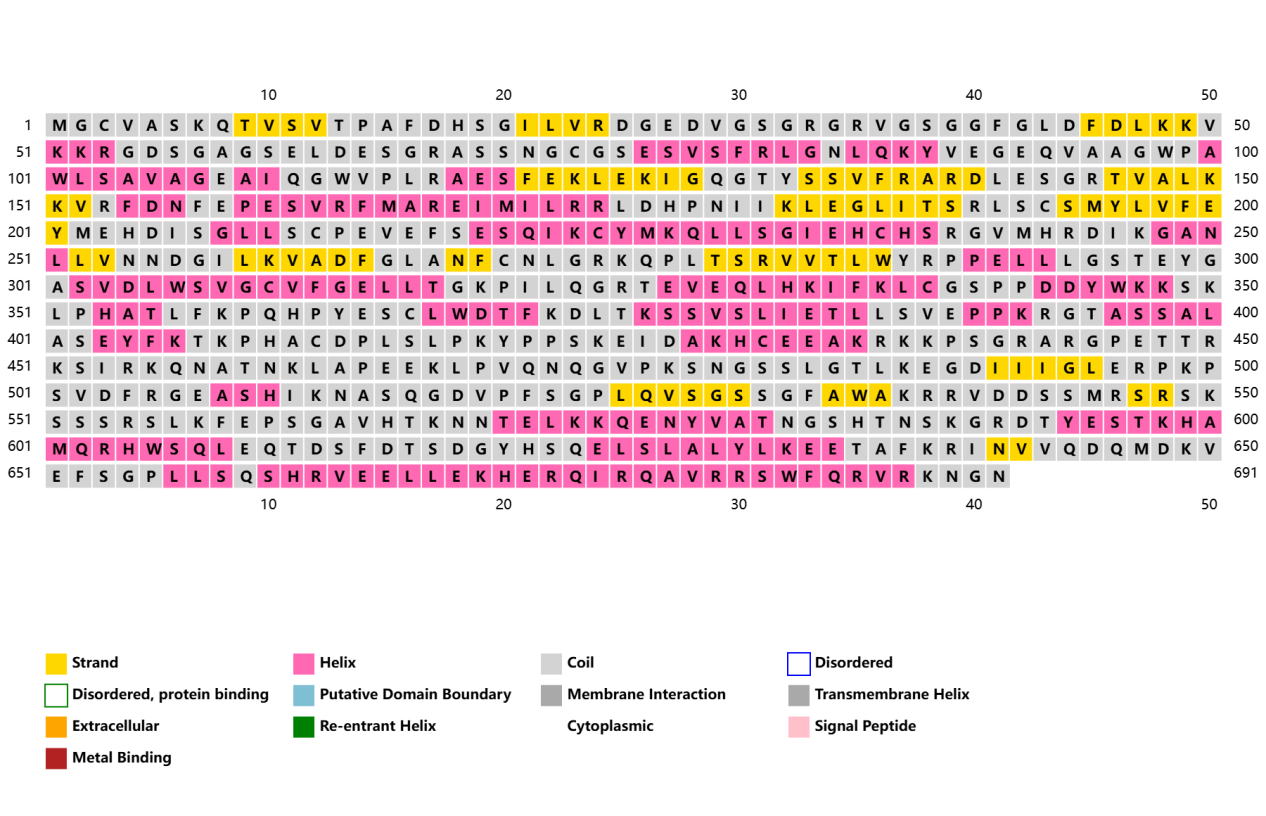
**

**Figure S9 The templates were found to match the target sequence.** (A) The template A0A6J1AFV5.1.A were found to match Sopen03g034650 amino acid sequence; (B) The template I1JZM1.1 were found to match Solyc03g115660 amino acid sequence.

**
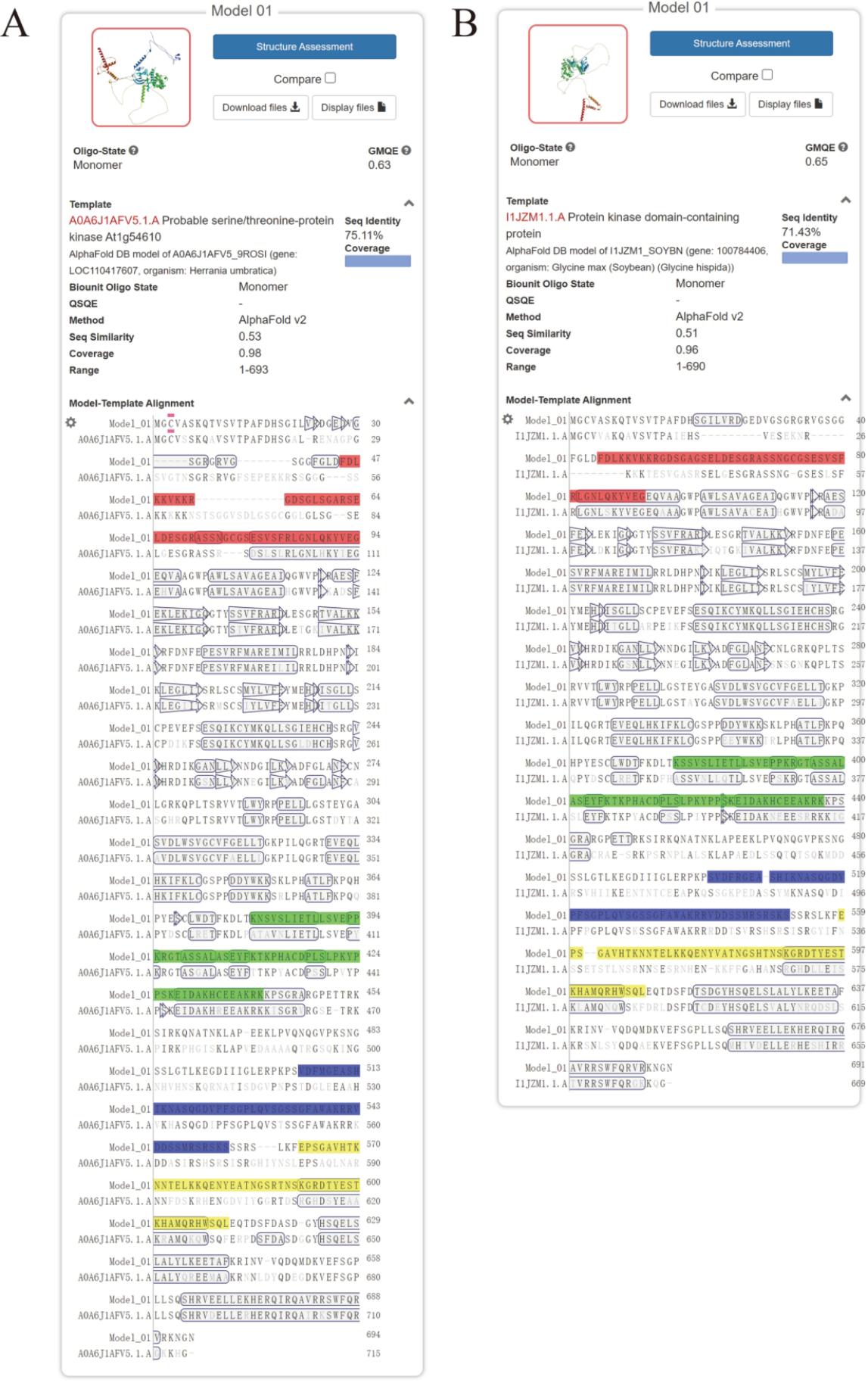
**


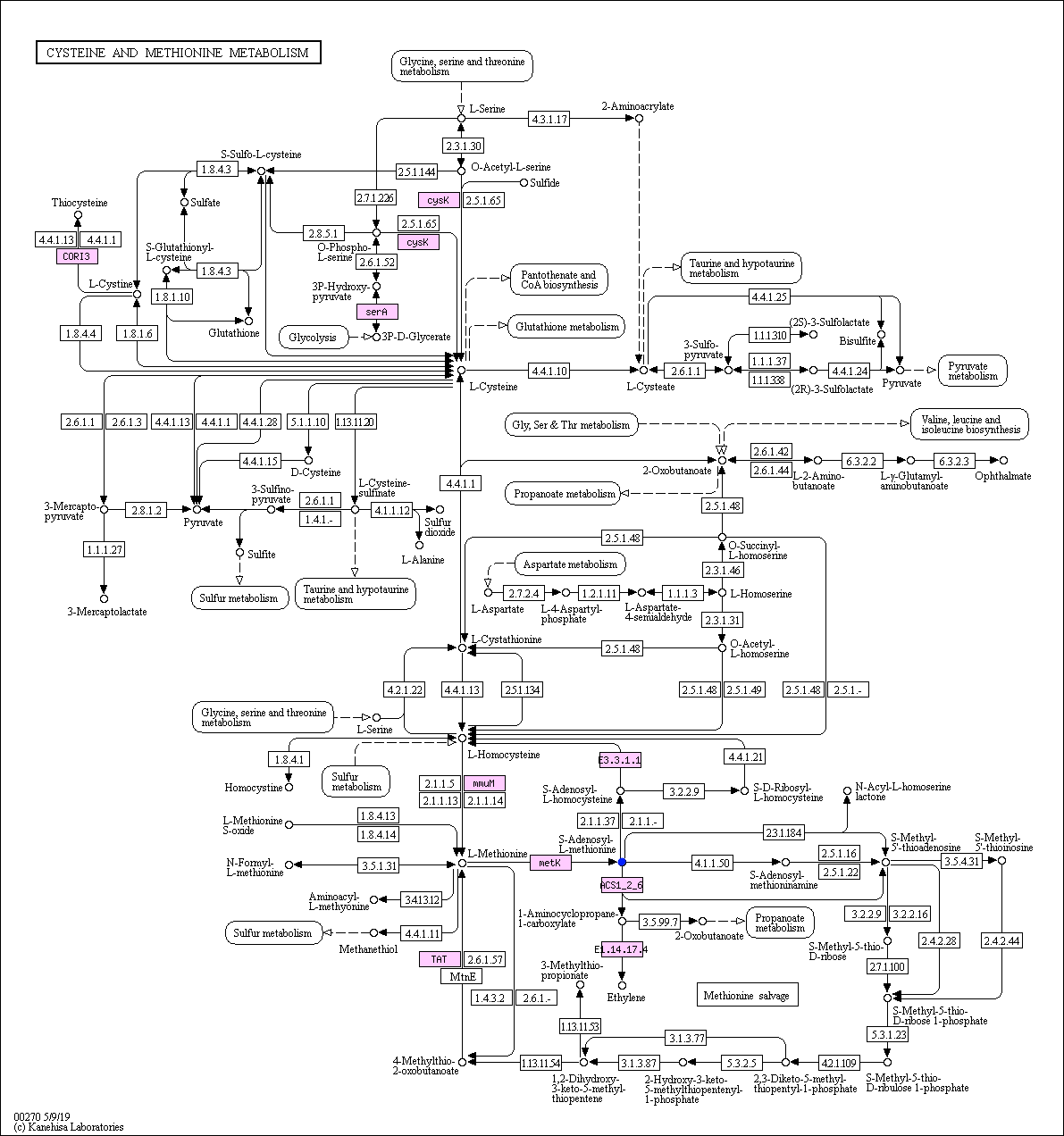
**Figure S10** The cysteine and methionine metabolism pathway plot identified by the integrated analysis of differentially expressed genes and differential accumulated metabolites from transcriptomic and metabolomic profiling at the mature green stage (blue dot represents downregulated).


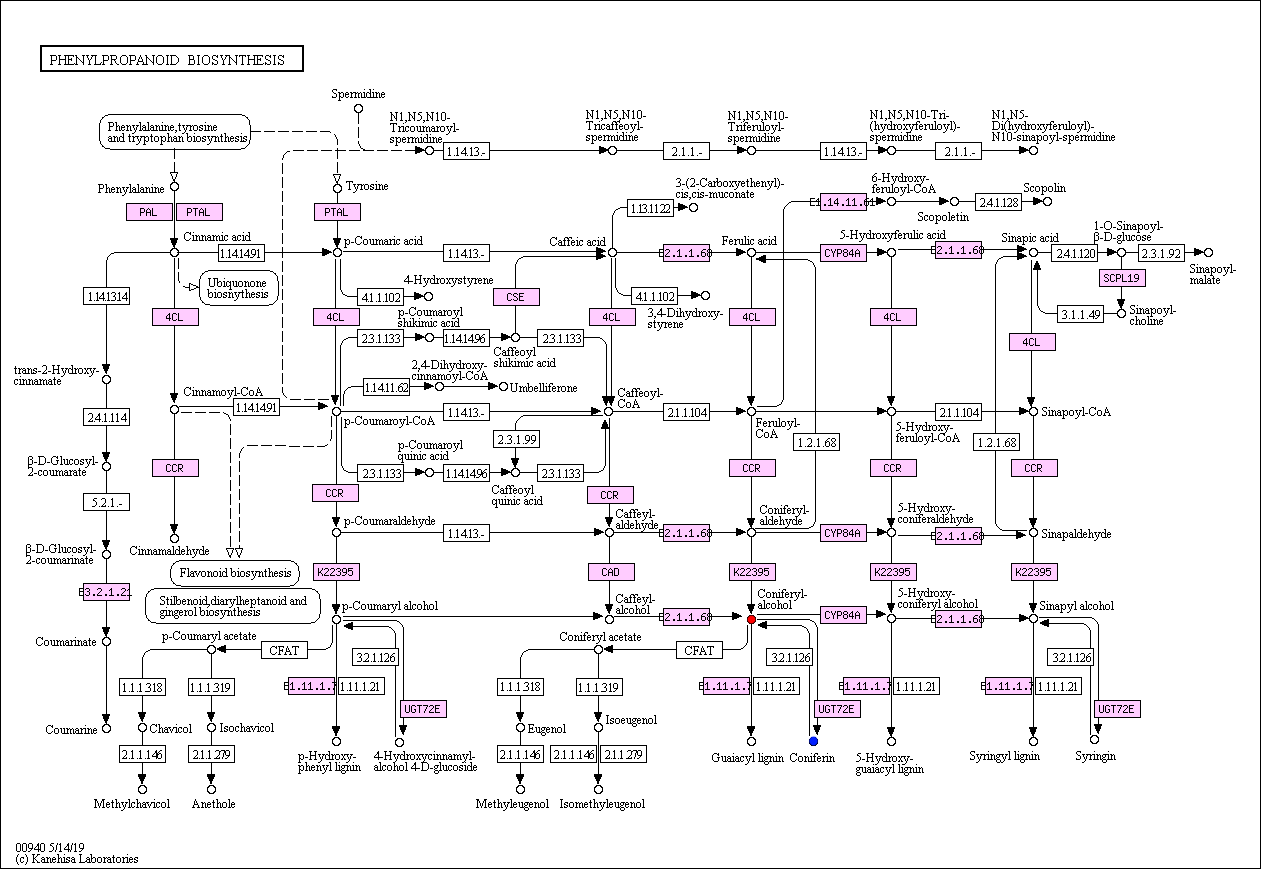
**Figure S11** The phenylpropanoid biosynthesis pathway plot identified by the integrated analysis of differentially expressed genes and differential accumulated metabolites from transcriptomic and metabolomic profiling at the mature green stage (red dot represents upregulated; blue dot represents downregulated).

**Figure S12** The phenylalanine, tyrosine and tryptophan biosynthesis pathway plot identified by the integrated analysis of differentially expressed genes and differential accumulated metabolites from transcriptomic and metabolomic profiling at the red ripe stage (red dot represents upregulated; blue dot represents downregulated).


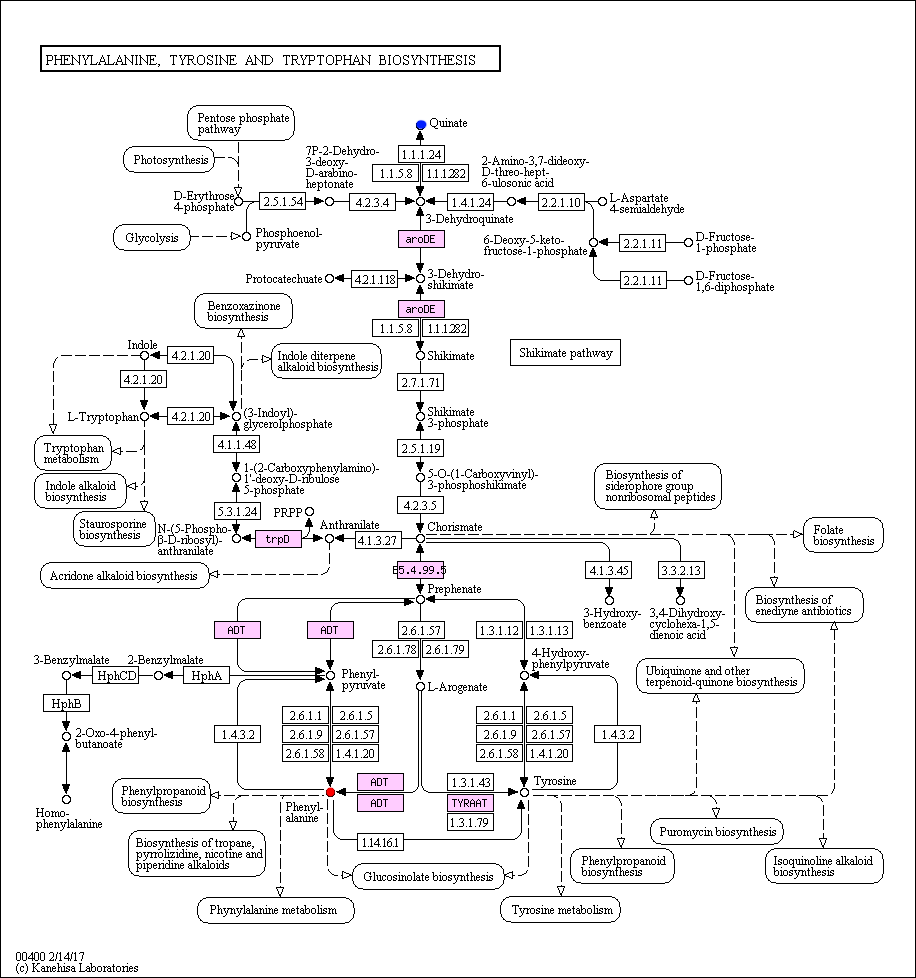


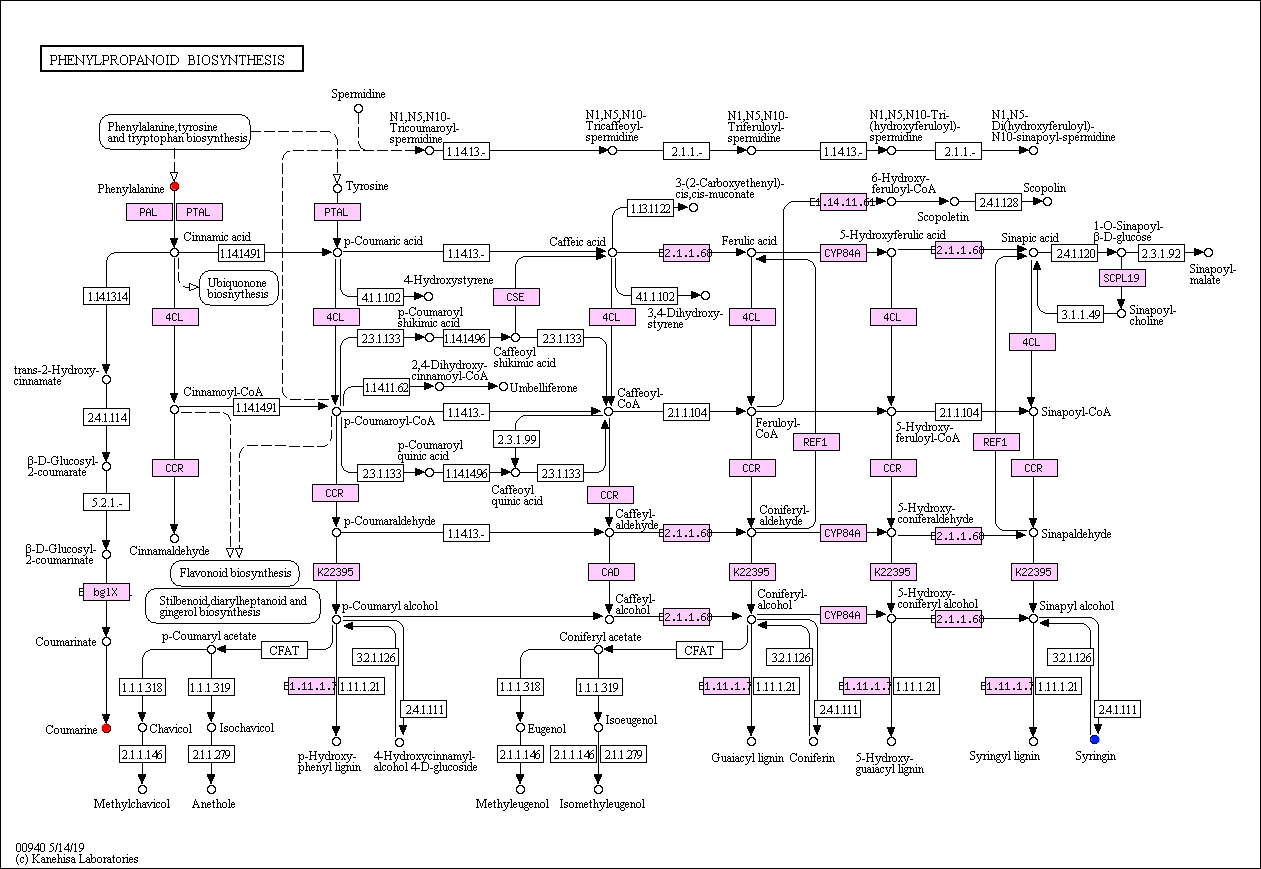
**Figure S13** The phenylpropanoid biosynthesis pathway plot identified by the integrated analysis of differentially expressed genes and differential accumulated metabolites from transcriptomic and metabolomic profiling at the red ripe stage (red dot represents upregulated; blue dot represents downregulated).

**Figure S14** The linoleic acid metabolism pathway plot identified by the integrated analysis of differentially expressed genes and differential accumulated metabolites from transcriptomic and metabolomic profiling at the red ripe stage (blue dot represents downregulated).


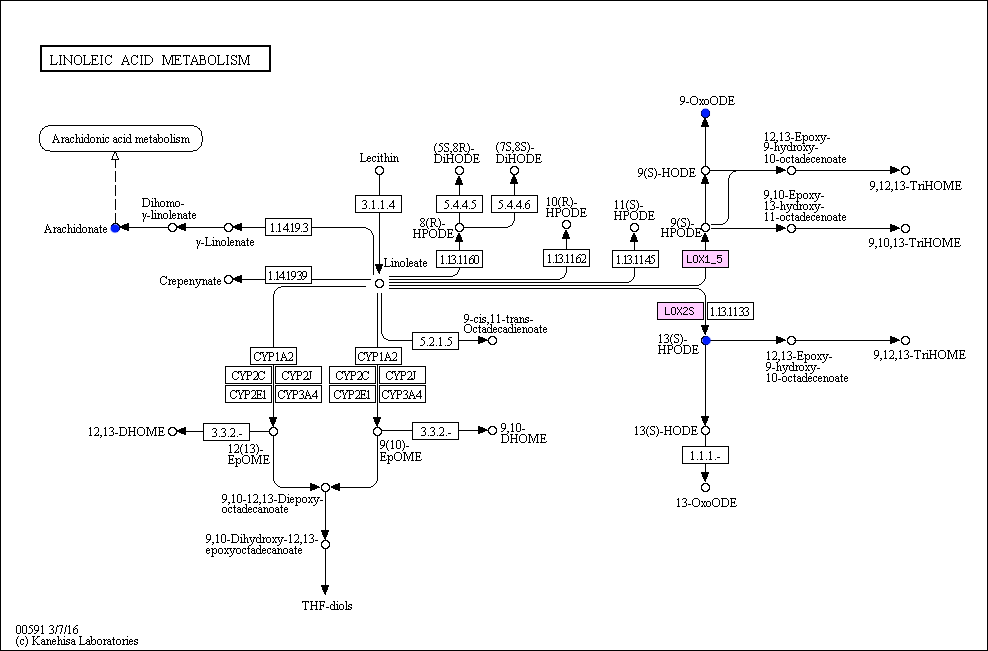

Supplement: Supplementary file 1 — Figure S1. Nucleotide sequences alignment of CDS between Sopen03g034650 and Solyc03g115660. Figure S2. Protein sequences alignment between Sopen03g034650 and Solyc03g115660. Figure S3. Nucleotide sequences alignment of CDS between Sopen03g034660 and Solyc03g115680. Figure S4. Protein sequences alignment between Sopen03g034660 and Solyc03g115680. Figure S5. The predicted signal peptides in the protein coded by the Cr3a gene. Figure S6. The predicted transmembrane domains on the protein coded by the Cr3a gene. Figure S7. Prediction of protein secondary structure of Sopen03g034650. Figure S8. Prediction of protein secondary structure of Solyc03g115660. Figure S9. The templates were found to match the target sequence. Figure S10. The cysteine and methionine metabolism pathway plot identified by the integrated analysis of differentially expressed genes and differential accumulated metabolites from transcriptomic and metabolomic profiling at the mature green stage (blue dot represents downregulated). Figure S11. The phenylpropanoid biosynthesis pathway plot identified by the integrated analysis of differentially expressed genes and differential accumulated metabolites from transcriptomic and metabolomic profiling at the mature green stage (red dot represents upregulated; blue dot represents downregulated). Figure S12. The phenylalanine, tyrosine and tryptophan biosynthesis pathway plot identified by the integrated analysis of differentially expressed genes and differential accumulated metabolites from transcriptomic and metabolomic profiling at the red ripe stage (red dot represents upregulated; blue dot represents downregulated). Figure S13. The phenylpropanoid biosynthesis pathway plot identified by the integrated analysis of differentially expressed genes and differential accumulated metabolites from transcriptomic and metabolomic profiling at the red ripe stage (red dot represents upregulated; blue dot represents downregulated). Figure S14. The linoleic acid metabolis [file TPJ-122-0-s001.docx]
